# Targeting TRPM3 as a potential therapeutic approach for autosomal dominant polycystic kidney disease

**DOI:** 10.1038/s41598-025-89200-z

**Published:** 2025-02-08

**Authors:** Hüseyin Gül, Jamie A. Davies

**Affiliations:** Deanery of Biomedical Sciences, University of Edinburgh, Hugh Robson Building, George Square, Edinburgh, UK

**Keywords:** Kidney diseases, Polycystic kidney disease, Pharmacology

## Abstract

Cystic diseases, especially autosomal dominant polycystic kidney disease (ADPKD; incidence approx. 1/1000), are a leading cause of renal failure, caused by appearance and growth of renal cysts that can lead to renal failure in middle age. Most ADPKD cases are caused by mutations in *PKD1* or *PKD2*, encoding polycystin-1 (PC1) and polycystin-2 (PC2). PC1 is a mechanosensor that controls PC2, a Ca^2+^-permeable cation channel that, by regulating cytoplasmic Ca^2+^, prevents adenylyl cyclase producing cyst-promoting concentrations of cAMP. In other systems, there is evidence that PC2 interacts with TRPM3. We therefore examined the effect of pharmacological activators and inhibitors of TRPM3 on cyst formation in cultured mouse kidney rudiments exposed to a range of concentrations of forskolin, a cAMP-elevating drug commonly used experimentally to induce cysts in cultured kidneys. We found that TRPM3 inhibitors (isosakuranetin, primidone, diclofenac) increased cyst formation, while TRPM3 activators (CIM0216 and nifedipine) greatly reduced cyst formation and reduced the sensitivity of kidneys to forskolin. These preclinical, in-vitro data suggest that TRPM3 may be a promising target in ADPKD management.

## Introduction

Autosomal dominant polycystic kidney disease (ADPKD) is the most prevalent lethal monogenic human Mendelian disorder, affecting 1 in 400–1000 people^1^. It is characterized by aberrant renal tubule structure and the progressive development and expansion of cysts within the kidneys. 50% of ADPKD patients over 60 years old develop end-stage renal failure^2^ and require dialysis or a kidney transplant. Treatment methods are limited. Surgically, renal cyst aspiration and laparoscopic cyst decortication can be used to remove individual large cysts. Medically, tolvaptan, a vasopressin 2 receptor (V2R) antagonist, and long-acting release octreotide (octreotide-LAR), a synthetic version of the natural hormone somatostatin, can slow disease progression, albeit with side-effects^3,4^. In addition to these approved medications, there are ongoing drug treatment studies targeting various cellular pathways including the MAPK pathway, NF-κB pathway, cell cycle, tyrosine kinase receptors, Na/K ATPase channels and epigenetic regulation mechanism (HDAC) for the treatment of ADPKD^5^.

Almost all cases of ADPKD are caused by mutations in one of two genes^6^: *PKD1* (about 78% of cases^7^) and *PKD2* (about 15% of cases^7^). These genes encode polycystin-1 (PC1) and polycystin-2 (PC2) proteins respectively. These proteins are found in the primary cilium of renal epithelial cells, and they act in a common pathway as well as showing some potentially independent activities^8^. PC1 is a large, multi-span transmembrane protein that seems to sense mechanical stimulation of the primary cilium, arising for example from fluid flow over the apical domain of the cell^9^. PC2 belongs to a subfamily of transient receptor potential (TRP) ion channels, and has the synonym TRPP2^10^ Four molecules of PC2 can associate as a homotetramer to produce a Ca^2+^-permeable nonselective cation channel^10^. Alternatively, PC2 can associate with other proteins to form heteromeric complexes. PC1 and PC2 form a heteromer in primary cilia and serve as a flow sensor activated by fluid flow changes in the lumen of renal tubules and induce Ca^2+^ influx through the PC2 channel^11^.

According to current understanding, the Ca^2+^-conducting activity of PC2-containing complexes is critical to the ability of wild-type PC2 to suppress development of renal cysts. Inflowing Ca^2+^ maintains a high enough concentration of cytoplasmic Ca^2+^ to suppress excessive activity of adenylate cyclase. There are two main pieces of evidence for this. One is that pharmacological stimulation of cAMP production, using cell-permeable analogues of cAMP (e.g.,^12^) or using activators of adenylate cyclase such as forskolin, drives cyst formation even in wild-type cells (e.g.,^13^). This can be seen in cells in 3D culture and also in kidney organoids or in embryonic mouse kidneys growing in culture (e.g.,^12,14,15^). The other piece of evidence is that cells carrying mutations in *PKD1* or *PKD2* show greatly enhanced sensitivity to cAMP elevation, showing more cysts than wild-type controls at the same concentration of cAMP-elevating drug, and requiring lower concentrations of these drugs to begin cyst formation at all. Cyst formation in response to elevated cAMP is driven by a combination of changes in cell polarity, changes in cell shape and convergent extension, and at least in the adult, aberrant cell proliferation^4^. Cells from cysts show an enhanced proliferative response to cAMP elevation, compared to normal adult cells, and consequent Erk pathway activation^16^. This enhanced sensitivity arises from their different cytoplasmic concentration of Ca^2+^^17^. As pointed out in^4^, though, proliferation is a natural feature of early nephron development (nephrons grow longer by a combination of proliferation and convergent extension) so cannot itself be sufficient for cyst formation in embryo-based assays.

The central role of cAMP in the model is the basis of the clinical use of Tolvaptan, which represses vasopressin-induced rises in cAMP, as a licensed drug for treatment of ADPKD patients^18^: the drug is not without its problems, which is why it is used only for highest risk patients (see^19^ for review).

PC2 can form complexes with other proteins. It can form 2:2 tetrameric complexes with transient receptor potential channel 1 (TRPC1), also expressed in renal epithelia on the plasma membrane^20,21^. Importantly, at least in simple cell assays, PC2 also forms complexes with transient receptor potential cation channel subfamily M member 3 (TRPM3)^20^. This association seems to be functionally very important: removing functional TRPM3 from cultured mIMCD3 kidney cells removes the PC2-dependent Ca^2+^ flux from these cells^20^. In other words, PC2 is not enough, even in the presence of PC1, to produce sufficient Ca^2+^ flux in this system: TRPM3 must be present as well. Cryo-EM evidence from HEK-293 cells engineered to produce tagged PC1 and tagged PC2 show that PC1 and PC2 can form a complex with a 1:3 stoichiometry^22^. The nature of this experiment does not exclude the possibility that, in cells that express TRPM3, PC1 and PC2 might also form heterotrimeric complexes with TRPM3 in place of one or more of the PC2 subunits. Alternatively, TRPM3 may be associated in some other way with the PC1/PC2 heterotetramer. The paper reporting the cloning of human TRPM3^23^ showed it to be expressed more strongly in kidney than anywhere else, based on Northern Blot evidence (central nervous system and testis were the only other sites showing clear evidence of expression). All of this suggests a hypothesis that TRPM3 might be a potential target in ADPKD. Testing that hypothesis is the focus of this report.

## Results

### Forskolin-driven cyst formation in cultured wild-type kidney rudiments

We chose an assay system based on cultured mouse embryonic kidney rudiments, that has been well-characterized and used by several laboratories in the past^12,24–29^. In this system, kidney rudiments are cultured in the presence of forskolin, which stimulates adenyl cyclase to raise intracellular concentrations of cAMP, or 8-Br-cAMP, which is a cell-permeable form of cAMP: the result is formation of locally greatly enlarged tubules. For brevity and in deference to established use^12,24–29^, we refer to these in the rest of this paper as ‘cysts’. It should be remembered, though, that these early expansions will not represent all of the features of cysts in the adult disease.

**Fig. 1:**
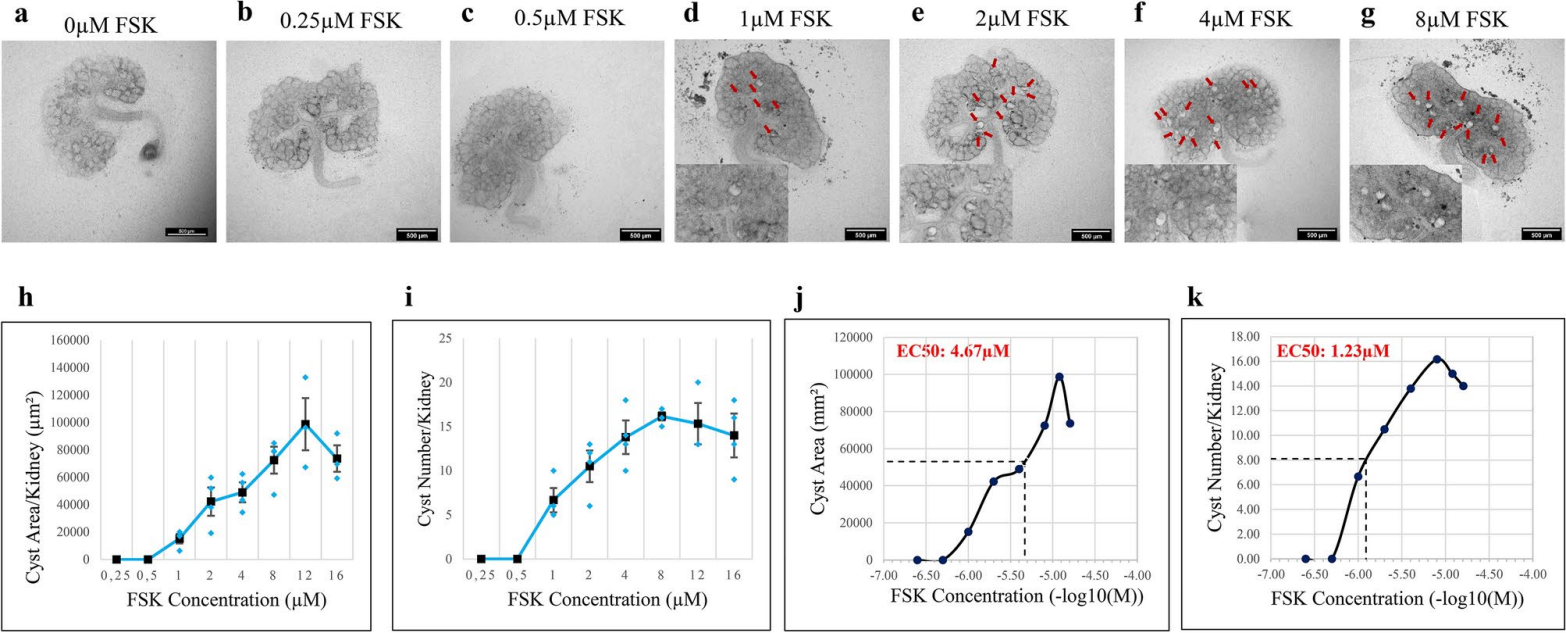
FSK induces cyst formation in a dose dependent manner in E12.5 kidney rudiments. Cultured E12.5 kidneys were treated with FSK and cyst formation was imaged and quantified after 2 days of culture. (**a**–**g**) Brightfield images of E12.5 kidney rudiments treated with 0 µM (untreated control), 0.25 µM, 0.5 µM, 1 µM, 2 µM, 4 µM and 8 µM FSK. **(h**,**i)** Quantification of cystic areas and cyst numbers in E12.5 kidney rudiments after 2 days of FSK treatment in increasing concentrations. (**j**,**k**) EC50 measurements for FSK from the number-of-cysts graph and the area-of-cysts graph. Cysts were indicated by red arrows. In (**h**) and (**i**), data are means of at least 3 kidneys and error bars indicate standard errors of the mean.

We cultured wild-type mouse embryonic day (‘E’) 12.5 kidneys, overnight (0.5 days) at an air–liquid interface in kidney culture medium (KCM: see Materials and Methods), then maintained them for a further 2 days in KCM supplemented with forskolin (FSK). Kidneys cultured in KCM alone, or with 0.25 µM or 0.5 µM FSK, showed no detectable cysts (Fig. 1a–c). However, cystic structures were observed at and above 1 µM FSK (Fig. 1d–g; red arrows). Larger images of Fig. 1b–g are provided in Fig. S1a–f, without annotation arrows. Total cyst area showed a quantitative dose/response curve (Fig. 1h), as did cyst numbers (Fig. 1i). The approximately sigmoidal nature of the curves allowed us to estimate the EC_50_ of FSK; for area, EC_50_ = 4.7 µM and for number, EC_50_ = 1.2 µM; Fig. 1j,k). For comparison, its EC_50_ value against adenyl cyclase itself on membrane preparations in vitro is in the range 0.5–1 µM^30^.

### TRPM3 inhibition potentiates FSK-driven cyst formation

The IUPHAR/ BPS Guide to Pharmacology^31^ lists sixteen inhibitors of TRPM3. Of these, isosakuranetin is the most potent; IC_50_ = 50 nM^32^, and has been used at 10 µM to inhibit the TRPM3 channel in mIMCD-3 renal cells^20^. Primidone (IC_50_ = 600 nM) and diclofenac (IC_50_ = 6.2 μM) are other reasonably potent reagents^33^ that are easy to obtain. We therefore used these to test whether inhibition of TRPM3 would promote cyst formation.

**Fig. 2:**
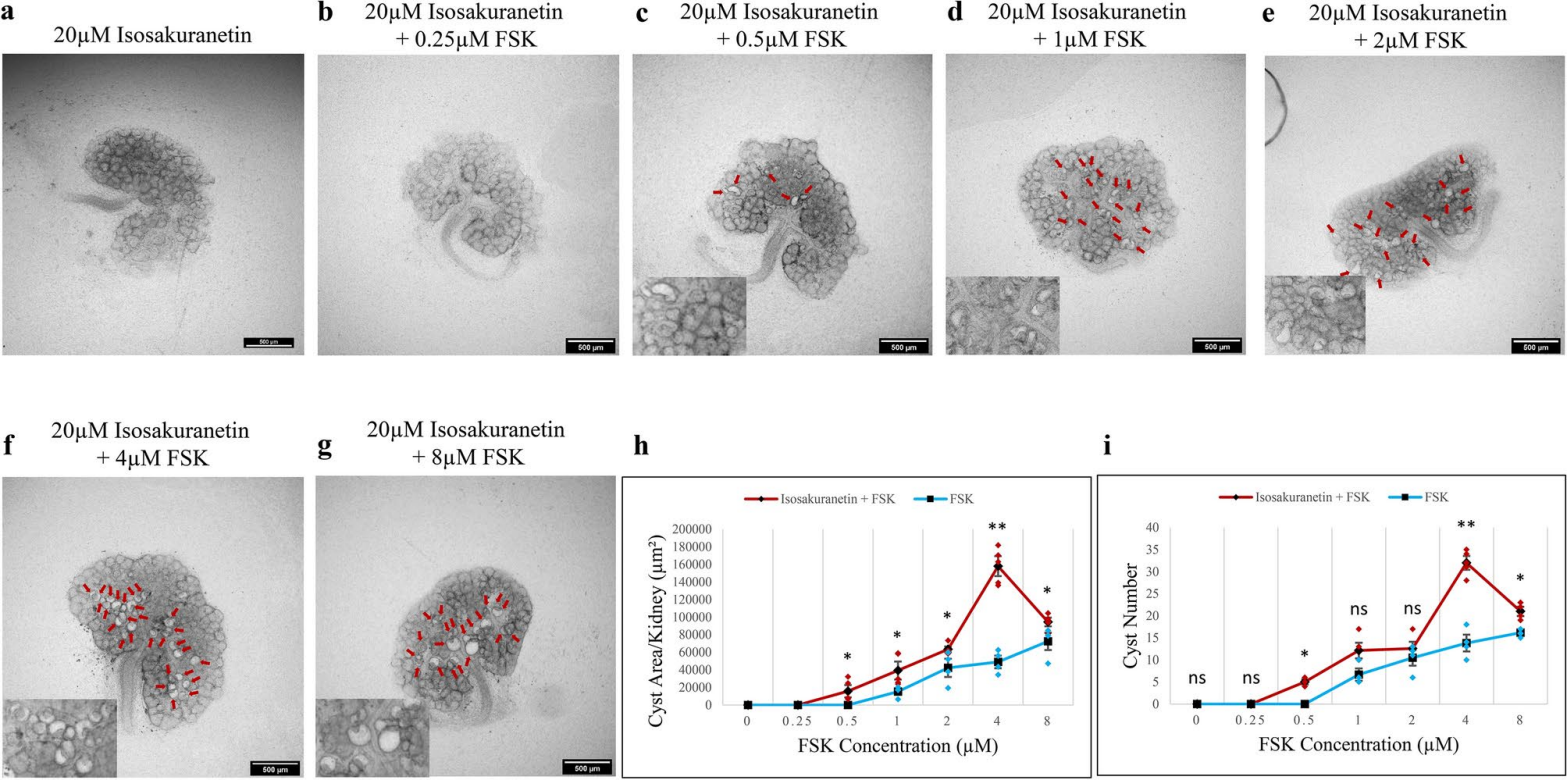
Isosakuranetin sensitized the kidneys to FSK, in terms of cyst formation. Cultured E12.5 kidneys were treated with 20 µM isosakuranetin and varying concentrations of FSK. Cyst formation was imaged and quantified after 2 days of culture. (**a**–**g**) Brightfield images of E12.5 kidney rudiments treated with 20 µM isosakuranetin and 20 µM isosakuranetin with 0.25 µM, 0.5 µM, 1 µM, 2 µM, 4 µM and 8 µM FSK. (**h**,**i**) Quantification of cystic areas and cyst numbers in E12.5 kidney rudiments after 2 days of 20 µM isosakuranetin with varying concentrations of FSK (maroon line) and FSK alone (blue line). Cysts were indicated by red arrows. In (**h**) and (**i**), data are means of at least 3 kidneys. Error bars indicate standard errors of the mean. *p*-values were calculated using unpaired t-tests. *p < 0.04, **p < 0.0002, ns; not significant. The FSK data presented are reproduced from Fig. 1.

Isosakuranetin (20 µM) alone did not induce cyst formation (Figs. 2 and S2 for larger images). This is not a surprise: FSK is required to stimulate cyst formation in culture even in *Pkd1* knock-out ex-vivo kidneys^12^. However, while 0.5 µM FSK did not induce cyst formation on its own (Figs. 1c and S1b for larger image), with isosakuranetin it did (Fig. 2c, red arrows; Fig. S2b for larger image) and higher FSK concentrations resulted in more and larger cysts (Fig. 2c–i, red arrows; Fig. S2 for larger images). In the absence of isosakuranetin, the number and area of cysts induced by different concentrations of FSK rises approximately linearly with the concentration, beyond 0.5 µM (Fig. 2h,i). With 20 µM isosakuranetin, the response peaks at 4 µM FSK, and then falls. At the peak, the increase in both cyst area and number are highly statistically significant (Fig. 2h,i; *p*-value < 0.0002). At the higher concentration of 8 µM FSK, the difference made by isosakuranetin was less pronounced, though still statistically significant (Fig. 2h,I; *p*-value < 0.04). No other abnormalities were observed in the kidney’s overall area or production of collecting duct branches or nephrons at any concentrations.

**Fig. 3:**
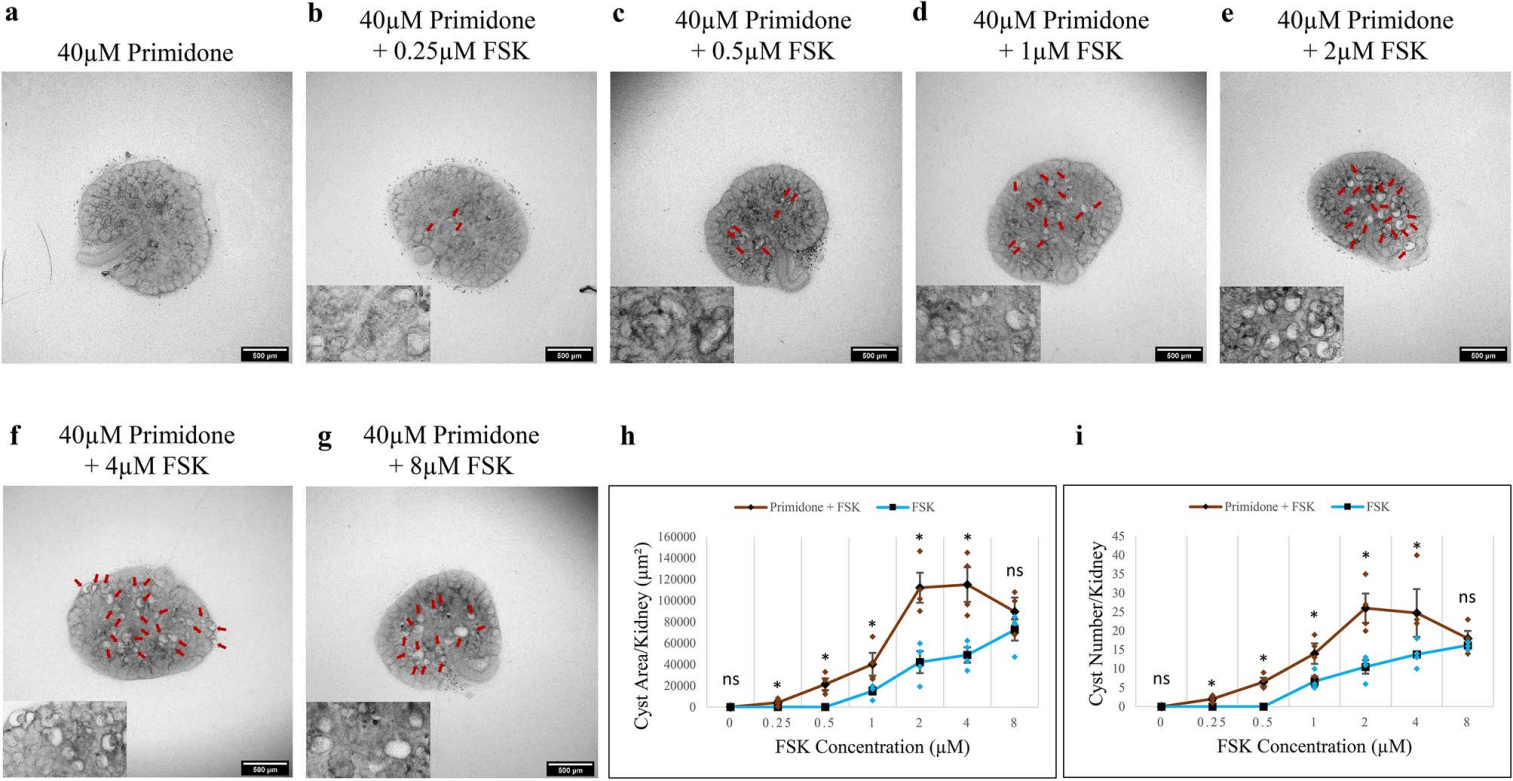
Primidone sensitized the kidneys to FSK, in terms of cyst formation. Cultured E12.5 kidneys were treated with 20 µM primidone and varying concentrations of FSK. Cyst formation was imaged and quantified after 2 days of culture. (**a**–**g**) Brightfield images of E12.5 kidney rudiments treated with 20 µM primidone and 20 µM primidone with 0.25 µM, 0.5 µM, 1 µM, 2 µM, 4 µM and 8 µM FSK. (**h**,**i**) Quantification of cystic areas and cyst numbers in E12.5 kidney rudiments after 2 days of 20 µM primidone with varying concentrations of FSK (red line) and FSK alone (blue line). Cysts were indicated by red arrows. In (**h**) and (**i**), data are means of at least 3 kidneys. Error bars indicate standard errors of the mean. *p*-values were calculated using unpaired t-tests. *p < 0.05, ns; not significant. The FSK data presented are reproduced from Fig. 1.

Primidone is a highly selective TRPM3 inhibitor, with no significant inhibition of other tested TRP channels (TRPV1, TRPM8, and TRPA1) at up to 50 µM^33^. We tested the effect of 40 µM primidone (just below the 50 µM of the just-mentioned study)^33^ on FSK-induced cyst development. No cysts formed in the absence of FSK (Fig. 3a), but small cystic structures were observed at FSK concentrations as low as 0.25 μM (Fig. 3b, red arrows; Fig. S3a for larger image). This is a concentration 4 times lower than the threshold FSK concentration in the absence of other drugs (Figs. 1d and S1c). Treated with 40 µM primidone and FSK, kidney rudiments showed a significant increase in both cyst area and number of cysts per kidney at all FSK concentrations (*p*-value < 0.05) except 8 µM (Fig. 3b–g, red arrows; Fig. 3h, i, brown line; Fig. S3a–f for larger images). As with isosakuranetin, 40 µM primidone resulted in the transformation of an essentially linear response to FSK into a distinct peak, between 2 and 4 µM, and an absolute fall beyond that, to come close to meeting the rising FSK-alone line at 8 µM, at which concentration there was no longer any significant difference (Fig. 3h,i). Peak cyst area and number per kidney were observed at 4 μM and 2 μM FSK concentrations, respectively, followed by a decrease at higher concentrations (Fig. 3h,i). Treatment of the kidney rudiments with the combination of 40 µM primidone and the highest FSK concentration (8 µM), did not make a significant difference to cyst area and in cyst number per kidney compared to the treatment of FSK alone (Fig. 3h,i).

**Fig. 4:**
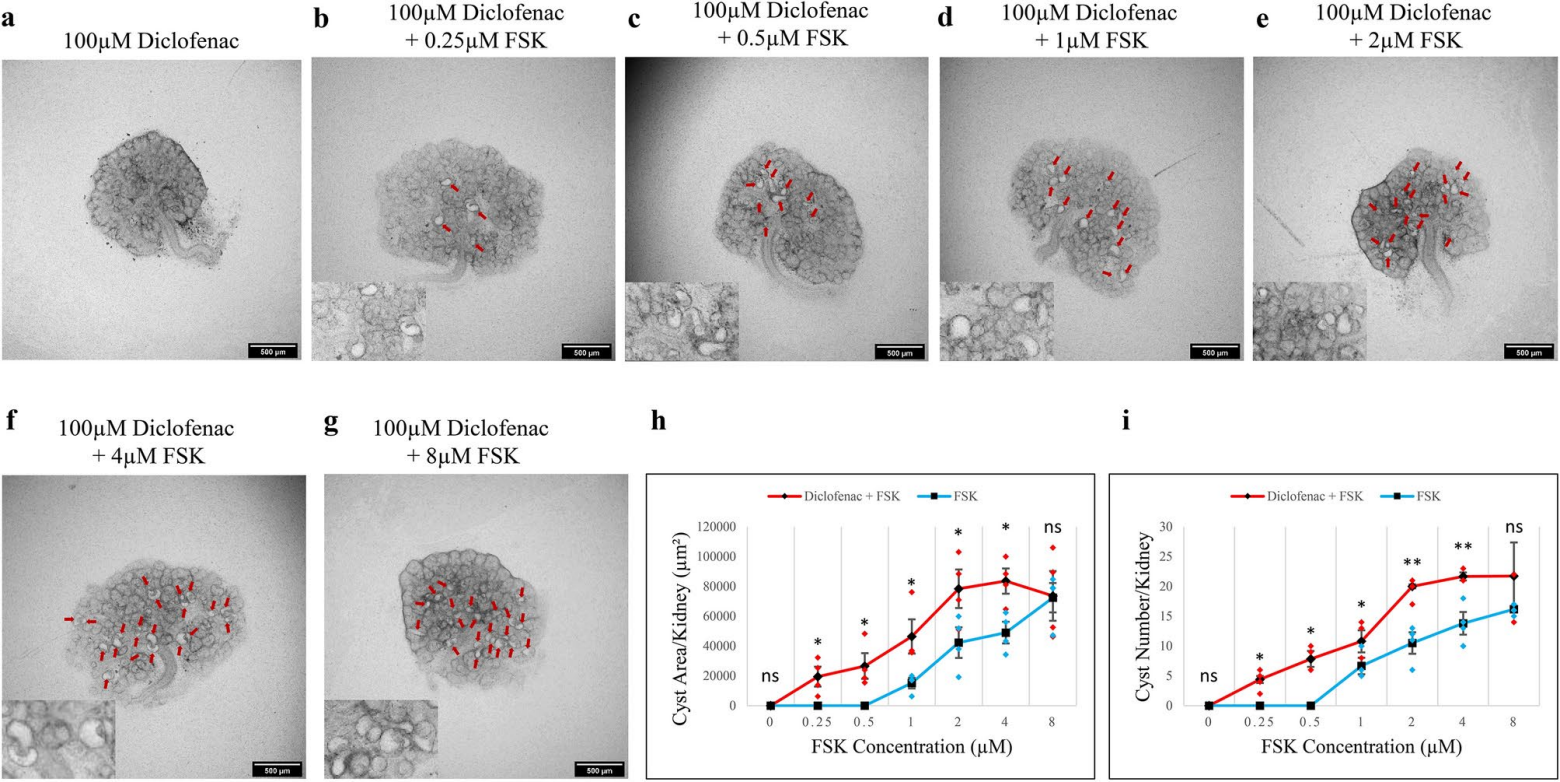
Diclofenac sensitized the kidneys to FSK, in terms of cyst formation. Cultured E12.5 kidneys were treated with 100 µM diclofenac and varying concentrations of FSK. Cyst formation was imaged and quantified after 2 days of culture. (**a**–**g**) Brightfield images of E12.5 kidney rudiments treated with 100 µM diclofenac and 100 µM diclofenac with 0.25 µM, 0.5 µM, 1 µM, 2 µM, 4 µM and 8 µM FSK. (**h**,**i**) Quantification of cystic areas and cyst numbers in E12.5 kidney rudiments after 2 days of 100 µM diclofenac with varying concentrations of FSK (brown line) and FSK alone (blue line). Cysts were indicated by red arrows. In (**h**) and (**i**), data are means of at least 3 kidneys. Error bars indicate standard errors of the mean. *p*-values were calculated using unpaired t-tests. *p < 0.03, **p < 0.001, ns; not significant. The FSK data presented are reproduced from Fig. 1.

Diclofenac, used as an analgesic as it inhibits COX1 and 2, is a third inhibitor of TRPM3^34,35^. Its targeting of COX-1 and COX-2 makes diclofenac a less clean reagent than isosakuranetin and primidone, but we felt it still worth comparing the effect of this reagent to the more specific inhibitors, especially because it is so widely prescribed. Cultured in FSK-free medium with the addition of 100 µM of diclofenac, kidneys developed normally with no sign of cyst formation (Fig. 4a). However, when the two reagents were used together, clear cystic structures formed even at 0.25 μM FSK (4 times lower than the threshold concentration in the treatment of FSK alone) (Fig. 4b, red arrows; Fig. S4a for larger image). As with primidone, diclofenac significantly elevated the number and area of cysts at all concentrations of FSK (Fig. 4b–g, red arrows; Fig. h, I, red line; Fig. S4a–f for larger images; *p*-value < 0.03) except 8 μM. And again, the area graph showed a distinct peak between 2 and 4 μM (in this region, *p*-value < 0.001), after which the response fell in absolute terms to meet the rising FSK-alone line. The graph of the number of cysts fell only very slightly between 4 and 8 μM (Fig. 4h,i).

**Fig. 5:**
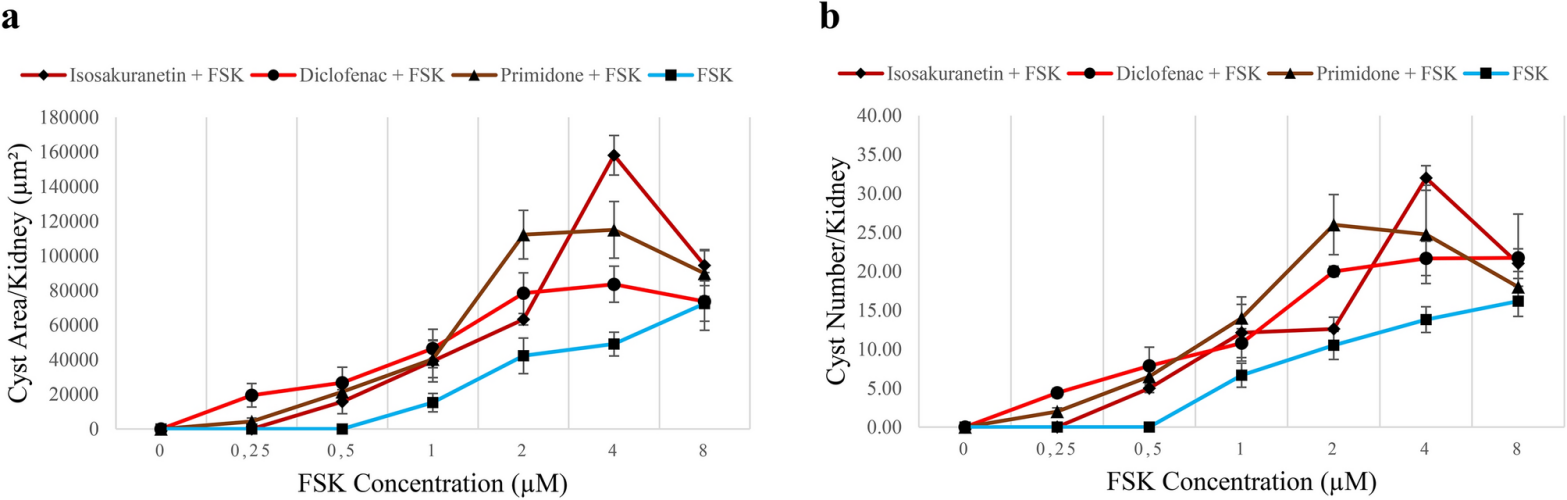
A comparison of the three TRPM3 blockers. Area-of-cysts graph and number-of-cysts graph of the kidneys treated with TRPM3 inhibitors alongside FSK (maroon line; isosakuranetin + FSK, brown line; primidone + FSK, red line; diclofenac + FSK) and FSK alone (blue line) were illustrated in (**a**) and (**b**) respectively. Error bars indicate standard errors of the mean. The data in these figures are the same data that are shown individually in Figs. 1, 2, 3 and 4.

A comparison of the three TRPM3 inhibitors, using data presented in Figs. 1, 2, 3 and 4, is shown in Fig. 5.

### TRPM3 activation inhibits FSK-driven cyst formation

**Fig. 6:**
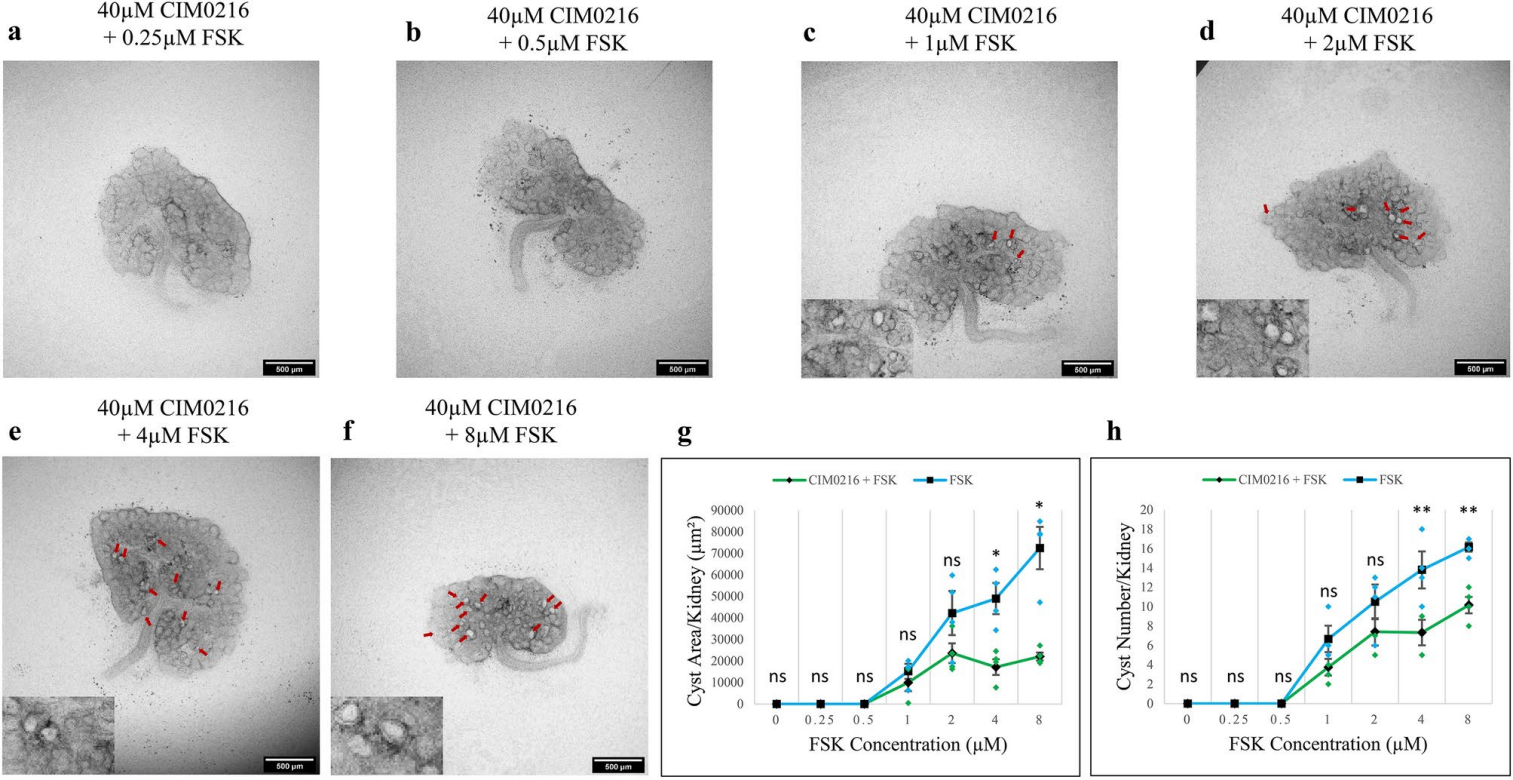
CIM0216attenuated FSK-driven cyst formation. Cultured E12.5 kidneys were treated with 40 µM CIM0216 and varying concentrations of FSK. Cyst formation was imaged and quantified after 2 days of culture. (**a**–**f**) Brightfield images of E12.5 kidney rudiments treated with 40 µM CIM0216 and 40 µM CIM0216 with 0.25 µM, 0.5 µM, 1 µM, 2 µM, 4 µM and 8 µM FSK. (**g**,**h**) Quantification of cystic areas and cyst numbers in E12.5 kidney rudiments after 2 days of 40 µM CIM0216 with varying concentrations of FSK (green line) and FSK alone (blue line). Cysts were indicated by red arrows. In (**g**) and (**h**), data are means of at least 3 kidneys. Error bars indicate standard errors of the mean. *p*-values were calculated using unpaired t-tests. *p < 0.002, **p < 0.004, ns; not significant. The FSK data presented are reproduced from Fig. 1.

Having established that TRPM3 inhibitors promote cyst development, we tested whether TRPM3 activators would have the opposite effect. We did this by using well-characterized TRPM3 activators, CIM0216 (EC_50_ = 0.8 μM in 2D cultured cells)^36^ and nifedipine (EC_50_ = 1.7 μM in 2D cultured cells)^37^. For 40 μM CIM0216, the threshold FSK concentration for cyst formation (1 μM) remained unchanged compared with FSK alone (Figs. 6a–c and S5a–c for larger images), but cyst formation, in terms of number and area, was reduced across all FSK concentrations capable of inducing cysts (Figs. 6c–f and S5c–f for larger images). At higher concentrations of FSK (4 μM and 8 μM) (Fig. 6g,h), this cyst-inhibiting effect of CIM0216 was highly significant for both cyst area (*p*-value < 0.002) and number (*p*-value < 0.004), the effect size being particularly marked in the case of area. Cyst area per kidney plateaued, or even showed a slight decrease, at FSK concentrations of 2 μM and above in the presence of CIM0216 (Fig. 6g). CIM0216 treatment resulted in a particularly dramatic effect at the highest FSK concentration, with cyst area reduced more than threefold compared to FSK alone (Fig. 6g). Additionally, cyst number per kidney exhibited a plateau (did not increase) between 2 and 4 μM FSK with CIM0216 treatment (Fig. 6h).

**Fig. 7:**
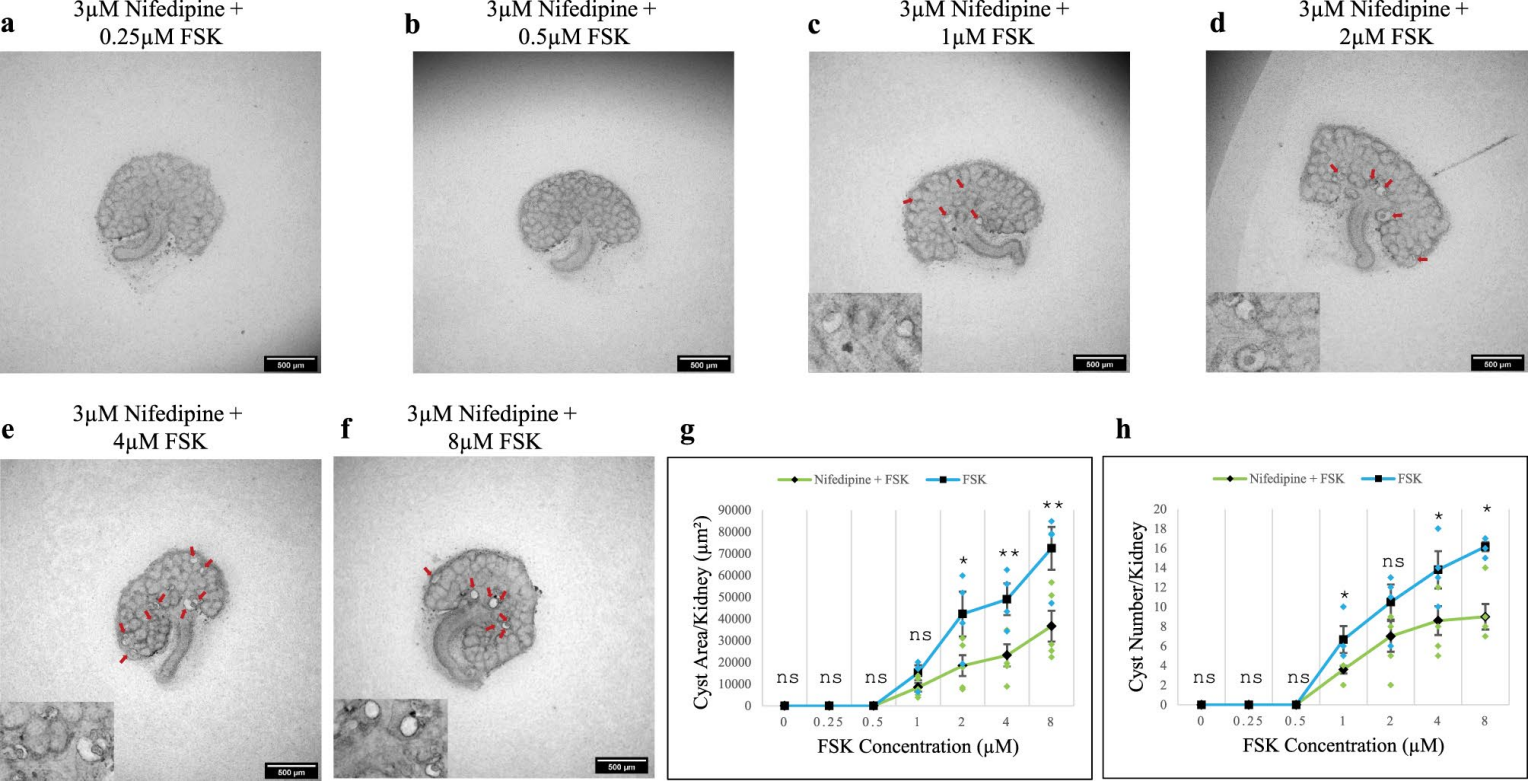
Nifedipine attenuated FSK-driven cyst formation. Cultured E12.5 kidneys were treated with 3 µM nifedipine and varying concentrations of FSK. Cyst formation was imaged and quantified after 2 days of culture. (**a**–**f**) Brightfield images of E12.5 kidney rudiments treated with 3 µM nifedipine and 3 µM nifedipine with 0.25 µM, 0.5 µM, 1 µM, 2 µM, 4 µM and 8 µM FSK. (**g**,**h**) Quantification of cystic areas and cyst numbers in E12.5 kidney rudiments after 2 days of 3 µM nifedipine with varying concentrations of FSK (green line) and FSK alone (blue line). Cysts were indicated by red arrows. In (**g**) and (**h**), data are means of at least 3 kidneys. Error bars indicate standard errors of the mean. *p*-values were calculated using unpaired t-tests. *p < 0.05, **p < 0.008, ns; not significant. The FSK data presented are reproduced from Fig. 1.

Nifedipine is used in the treatment of high blood pressure and Raynaud’s syndrome. It primarily targets L-type Ca^2+^ channels, inhibiting them, but also it happens activate TRPM3 with moderately high affinity^37^. Although TRPM3 is not its primary target, it was worth examining the effect of nifedipine on cyst formation in our system, as L-type Ca^2+^ channels are not expressed in the nephrons or collecting ducts of kidneys at the early stages of development being studied here, according to transcriptomic data in the www.gudmap.org database of gene expression in kidney development. This avoids a conflating effect that would be present in adult kidneys, and provides a second TRPM3 activator with a structure very different from CIM0216 and therefore unlikely to have the same off-target effects. The threshold FSK concentration for cyst formation (1 µM) did not change with 3 µM nifedipine treatment (Figs. 7a–c and S6a–c for larger images), as in the CIM0216 trial, but there was a noticeable reduction in cyst formation (both in cyst area and cyst number) at all FSK concentrations (Figs. 7c–h and S6c–f for larger images). Although cyst formation gradually increased with higher FSK concentrations in the presence of nifedipine, this increase was lower compared to kidneys without nifedipine (Fig. 7g, h). This effect was even strongest at higher FSK concentrations (4 μM and 8 μM) in cyst area (*p*-value < 0.008) and cyst number (*p*-value < 0.02) (Fig. 7g, h).

## Summary of TRPM3 activator and inhibitor data

**Fig. 8:**
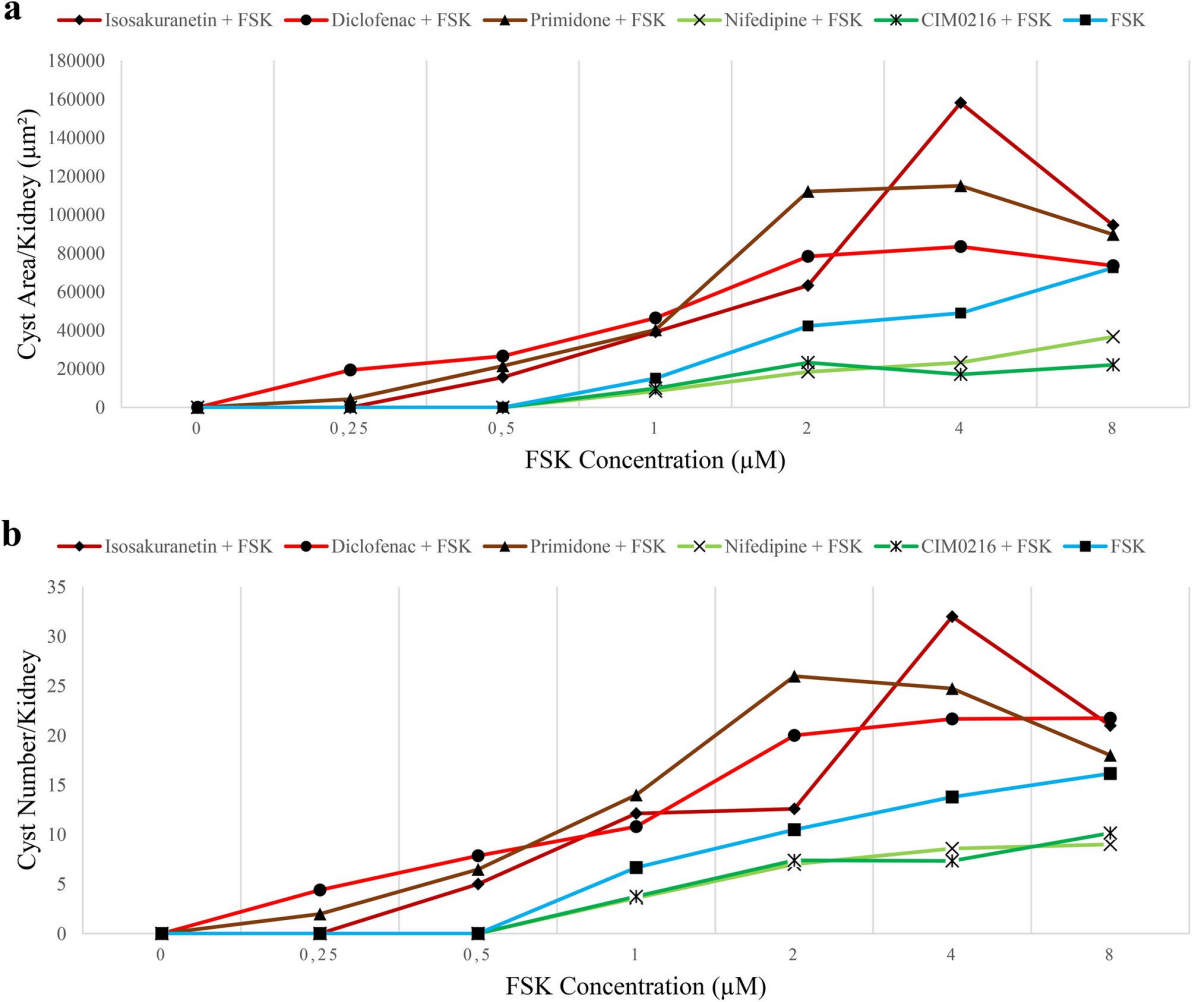
Summary of TRPM3 activator and inhibitor data. Area-of-cysts graph and number-of-cysts graph of the kidneys treated with TRPM3 inhibitors alongside FSK (maroon line; isosakuranetin + FSK, brown line; primidone + FSK, red line; diclofenac + FSK), TRPM3 activator alongside FSK (green line) and FSK alone (blue line) were illustrated in (**a**) and (**b**) respectively. Error bars indicate standard errors of the mean. The data in these figures are the same data shown individually in Figs. 1, 2, 3, 4, 6 and 7.

Figure 8 brings together the data already described in the above sections, to allow the effects of all of these drugs to be compared directly. The blue line shows the dose–response curve to FSK alone. Consistent with our hypothesis, TRPM3 blockade by isosakuranetin, primidone and diclofenac (red/brown lines) increased number and areas of cysts, while TRPM3 activation by CIM0216 and nifedipine (green lines) led to a marked attenuation of the cyst-promoting activity of FSK.

### TRPM3 modulation impacts cyst formation driven by IBMX

To confirm whether the effect of TRPM3 modulators on cyst formation was cAMP-dependent, we cultured E12.5 wild-type mouse kidneys immediately after dissection in KCM supplemented with TRPM3 modulators (isosakuranetin and nifedipine) along with varying concentrations of IBMX (3-isobutyl-1-methylxanthine). This is a nonselective inhibitor of cAMP and cGMP phosphodiesterases that raises cAMP by retarding its degradation. To establish a baseline for normalizing the effect of TRPM3 modulation on cyst formation in kidneys with blocked cAMP degradation by IBMX, we first generated a plot of cyst formation in kidneys treated with varying concentrations (0.5–50 µM) IBMX alone for 5 days.

Kidneys cultured in KCM with 0.5 µM, 1 µM, and 5 µM IBMX showed no detectable cyst formation (Fig. S7a–c). Cystic structures (red arrows) appeared at 10 µM IBMX and became very obvious at 25 and 50 µM (Fig. S7d–f). Quantitative analysis of total cyst area revealed a dose-dependent increase between 10 µM and 50 µM IBMX (Fig. S7g). Similarly, counts of cyst numbers in 2D images followed a dose/response trend at the same concentrations of IBMX (Fig. S7h). IBMX concentrations of 75 µM and above were found to be toxic to the kidneys (data not shown). It is also worth noting that IBMX stimulated cyst formation more slowly than FSK (4 days vs. 1 day). This may reflect the fact that FSK works by increasing cAMP production, IBMX works by reducing cAMP destruction and cAMP accumulates only at its natural rate of synthesis.

In E12.5 kidneys treated with the TRPM3 inhibitor, isosakuranetin, along with a range of IBMX concentrations, cyst formation was observed at all tested IBMX concentrations from 0.5 to 50 µM (Fig. S8a–f). Cyst formation in these kidneys was higher than in those treated with IBMX alone; except at 50uM in the cyst area plots and 25 uM in the cyst number plots, all comparisons were significantly different at *p* < 0.04. (Fig. S8g,h). A marked increase in cyst formation was evident at IBMX concentrations above 10 µM (Fig. S8g,h).

In E12.5 kidneys treated with nifedipine along with a range of IBMX concentrations, cyst formation was repressed (Fig. S9a–f). A significant reduction in cyst area was observed in kidneys treated with nifedipine and IBMX at 25 µM and 50 µM compared to kidneys treated with IBMX alone at the same concentrations (Fig. S9g; *p*-value < 0.04). The effect on cyst number was in the same direction, but larger variations, but the differences do not pass a test for significance; i.e., *p* > 0.05 (Fig. S9h). A comparison of findings from IBMX-driven cystic kidneys clearly demonstrated that TRPM3 inhibition with isosakuranetin sensitized the kidneys to IBMX-induced cyst formation, increasing both cyst area and number (Fig. S10a,b). In contrast, TRPM3 activation significantly reduced the cystic area in IBMX-treated kidneys (Fig. S10a). These results in IBMX-driven cystic kidneys strongly support the idea that TRPM3 impacts cyst formation through a pathway involving cAMP signaling.

## Discussion

Our results show that the cyst-promoting effect of cAMP elevation on developing mouse kidneys is exacerbated by co-incubation with any of a range of TRPM3 inhibitors, but is attenuated by co-incubation with TRPM3 activators.

Our work was stimulated by the possibility that TRPM3 cooperates with PC2, perhaps as a heterotetramer, as has been suggested by observations in a mouse renal cell line^20^, to regulate influx of Ca^2+^ ions and thereby modulate the activity of adenylyl cyclase. Our results in FSK-driven tubule expansion are compatible with that idea and the findings in IBMX-driven tubule expansion provide strong evidence that TRPM3 modulation impacts cyst formation via a pathway that involves cAMP signaling.

Establishing whether TRPM3 forms heteromers with PC2 in kidney cells would require quite different techniques, such as FRET between one fluorescent label on TRPM3 and another on PC2 (as done for detecting other heteromeric complexes in^38,39^), or cryo-EM, or immuno-gold electron microscopy using different sized of gold particles on each antibody (use for detecting heteromeric receptors is reviewed by^40^). Establishing whether the effect of modulating TRPM3 does alter cytoplasmic Ca^2+^ level could be done by using fluorescent reporters of Ca^2+^ concentration such as Fura-2^41^. To obtain further evidence that TRPM3 affects cyst formation via cAMP signaling, fluorescent reporters of cAMP concentration such as G-Flamp2^42^ could be employed. If it were all done, this work would provide valuable information about mechanism, but detailed knowledge of this mechanism is not essential for exploring the medical potential of our discovery. If TRPM3 activators antagonize cyst formation, this may be therapeutically useful whatever the molecular link between TRPM3 and cyst formation.

The findings we present here are a starting point for work towards human therapy, but it is important to note that a considerable amount of pre-clinical work needs to be done before clinical trials would be appropriate. Our work used mouse tissue, and it will be important to check that the same effects are visible in equivalent human tissues, for example renal organoids made from pluripotent human cells^43–45^. Such organoids have been used to model ADPKD^46^. Our work used forskolin and IBMX to induce cysts, rather than genetic mutation of polycystin genes. Even polycystin mutants require forskolin, or cAMP analogues, to produce cysts efficiently in typical in vitro assays^47–49^ although some recent long-duration organoid protocols using knockout cells produce a reasonable frequency of cysts in their absence^50^. Time may be an important factor: most in vitro assays last days but the human disease takes decades to develop^51^. It would, nevertheless, be very useful to discover whether TRPM3 activation makes polycystin mutants relatively resistant to forskolin-induced cyst formation, ideally restoring it to the lower sensitivity of wild-type. It would also be very useful to discover whether TRPM3 activation reduces forskolin-induced cyst formation in PC2 mutants as well as in the more common PC1 mutants. One of the ways that wild-type PC1 inhibits cyst formation is its activation of Ca^2+^ flux via PC2. Promoting a Ca^2+^ flux via TRPM3 would therefore be expected to ameliorate the effects of function-blocking mutations in PC1. If TRPM3 were independent of PC2, it may also ameliorate PC2 mutation but, if TRPM3 forms a heteromeric channel with PC2, TRPM3 activators may not result in significantly elevated Ca^2+^ flux when in a heteromeric channel with a mutant PC2. This would be only a small limitation to therapeutic application; most cases of ADPKD are mutations in the gene encoding PC1^52^. Naturally, it would also be important to use a long-term organoid assay that does not require pharmacological elevation of cytoplasmic cAMP (e.g.^50^).

The other important difference between our system and the real disease is that our work used early kidney rudiments, not mature tissue undergoing the slow process of cystic transformation that characterizes human ADPKD. Mouse models for ADPKD do exist, both natural and transgenic^53^. Even mutants of *PKD1* and *PKD2* analogous to those found in humans, the mouse models do not model the human disease exactly (reviewed by^54,55^); heterozygotes of the *pkd1* gene, encoding PC1, show slow or absent cystic disease, though they have an inappropriate antidiuretic phenotype^56^ while homozygotes show ultra-rapid lethality in the first weeks of life. Delayed inactivation of the gene, so that it is deleted only in adult life, results only in a mild cystic phenotype^57^. It is therefore not clear how useful trials in the mouse model would be in the path to clinical trials, if results from the mutant human organoid experiments outlined in the paragraph above were positive.

Our aim, in this work, was to explore a possible therapy to block cyst initiation and progression. There is evidence from mouse models that reinstatement of PC1 and PC2 expression, after their suppression caused development of early ADPKD, reverses the disease at least at a gross phenotypic level^58^. This raises the possibility that imposing control over the cystogenic phenotype by TRPM3 activators may be useful even in patients in whom the disease has begun. This is something that might be addressable in animal models.

## Materials and methods

### Embryonic kidney culture

All procedures listed here were approved by the Institutional Animal Care and Use Committee of the University of Edinburgh, and were carried out in accordance with their guidelines and regulations. All methods were reported in accordance with ARRIVE guidelines. All animals (CD1 mice) were obtained from Charles River Laboratories.

Pregnant CD1 mice were culled at E12.5 by methods listed under Schedule 1 of the UK Animals Scientific Procedures Act 1986, by trained animal house staff licensed by the UK Home Office. The morning of vaginal plug detection was considered E0.5. Metanephric kidney rudiments were dissected from E12.5 embryos and cultured on 0.4 µm-pore polyester membranes (Trans wells, Corning 3460) in kidney culture medium (KCM: MEM [Gibco 10370021], with 10% FBS [Gibco A5256801], and 1% penicillin/streptomycin [Gibco 15070063]). Kidneys were incubated overnight (0.5 days) then maintained for a further 2 days in either plain KCM or in KCM supplemented with modulators of adenyl cyclase or TRPM3 (see ’reagents’ section below for details). Control kidneys were administered the amount of DMSO at the highest concentration of the reagent(s) used in that experiment. In combined treatments (FSK + TRPM3 agonist or antagonist), the total amount of DMSO at the highest FSK concentration was used. After 2 days treatment, bright-field images of the kidneys were taken by using a Nikon Ti2 Inverted microscope (Plan Apo λ 4 × MRD00045).

### Assignment of Kidneys to Experimental Groups

The kidneys were dissected from E12.5 embryos and pooled by the dissector before being randomly assigned to different treatment groups. Results for each experimental group, (e.g., FSK alone, FSK + Isosakuranetin, FSK + Primidone, etc.) were obtained from two pregnant CD1 mice (n = 2), excluding any losses due to technical issues. In total, 25 pregnant CD1 mice (n = 25) were utilized for this study, including those lost during the experiments due to technical challenges such as contamination of cultured kidneys.

### Identification of cystic structures

Cystic structures were identified based on the following three characteristics: appearing brighter and being larger in diameter than the tubule, having two borders surrounding the lumen area and cyst area, and having smooth round morphology. Structures exhibiting at least two of these features were classified as cysts and included in the quantification graphs.

### Reagents

Isosakuranetin (PHL82569) was purchased from Merck, forskolin (B1421), primidone (B2120) and nifedipine (B1988) from ApexBio, diclofenac (HY-15036-1 mL) from Cambridge Bioscience and CIM0216 (27684) from Cayman Chemical, IBMX (72762) from StemCell Technologies. Isosakuranetin, CIM0216 and IBMX were prepared as 10 mM stock solutions in DMSO. Other reagents were obtained as a 10 mM stock solution in DMSO.

### Statistical analysis

Cyst areas of the cystic kidneys were selected by manually drawing round them and measured using ImageJ software. Values are shown as the mean ± standard error of at least three experiments. Comparisons between each experimental sample and its control, or between two different experimental samples, were analyzed by unpaired t-test using the GraphPad Prism t-test calculator tool. Values of *p* < 0.05 were considered as statistically significant.

## Supplementary Information

Supplementary Legends.

Supplementary Figures.

## Data Availability

The data that support the findings of this study are openly available at the following URL/DOI: 10.7488/ds/7861.
