# Correction: Targeting TRPM3 as a potential therapeutic approach for autosomal dominant polycystic kidney disease

**DOI:** 10.1038/s41598-025-09561-3

**Published:** 2025-07-15

**Authors:** Hüseyin Gül, Jamie A. Davies

**Affiliations:** Deanery of Biomedical Sciences, University of Edinburgh, Hugh Robson Building, George Square, Edinburgh, UK

Correction to: *Scientific Reports* 10.1038/s41598-025-89200-z, published online 08 February 2025

The original version of this Article contained an error in Figure 7, panels g and h, where the labels mistakenly repeated those from Figure 6. The original Figure 7 and accompanying legend appear below.

**Fig. 7:**
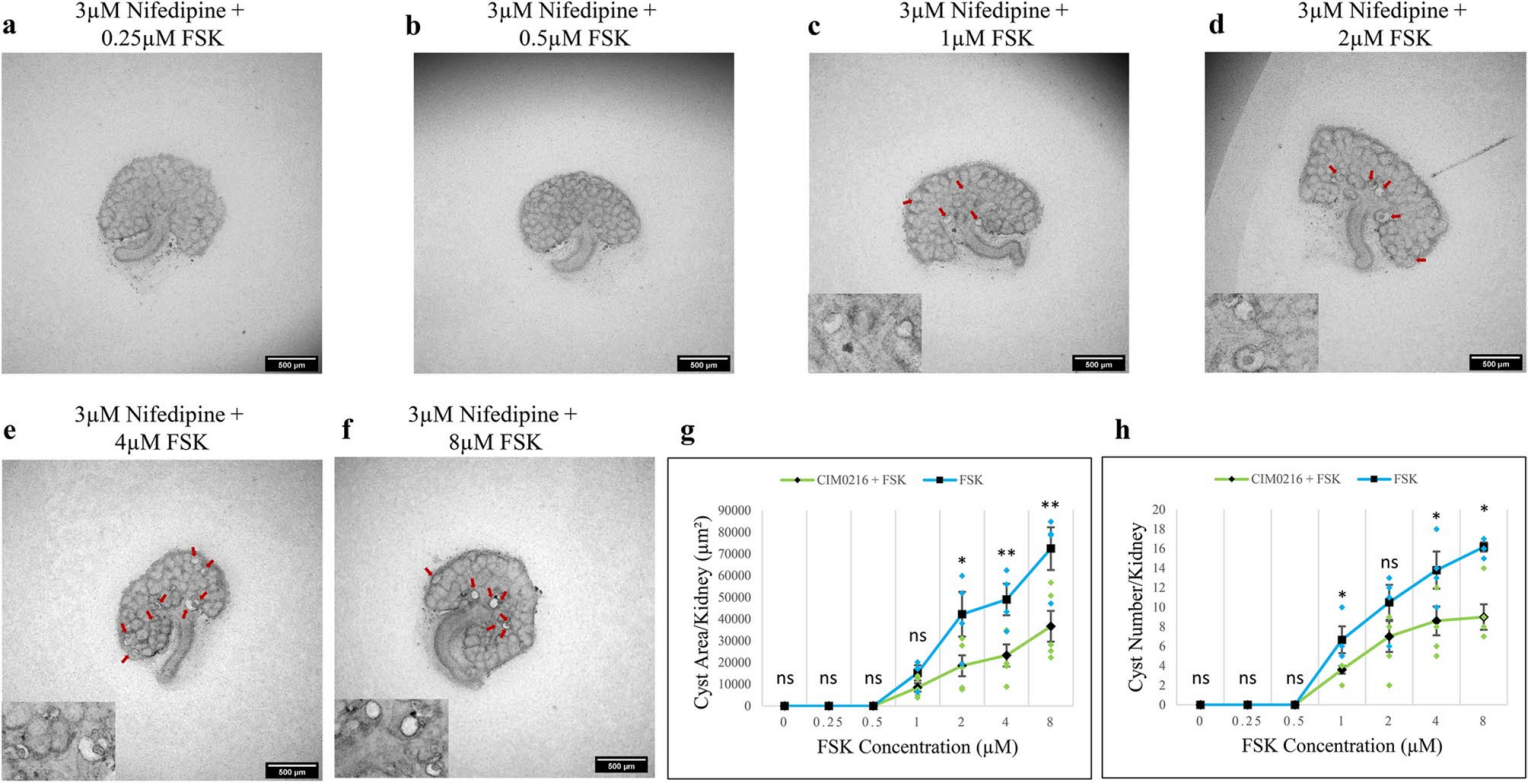
Nifedipine attenuated FSK-driven cyst formation. Cultured E12.5 kidneys were treated with 3 µM nifedipine and varying concentrations of FSK. Cyst formation was imaged and quantified after 2 days of culture. (**a**–**f**) Brightfield images of E12.5 kidney rudiments treated with 3 µM nifedipine and 3 µM nifedipine with 0.25 µM, 0.5 µM, 1 µM, 2 µM, 4 µM and 8 µM FSK. (**g**,**h**) Quantification of cystic areas and cyst numbers in E12.5 kidney rudiments after 2 days of 3 µM nifedipine with varying concentrations of FSK (green line) and FSK alone (blue line). Cysts were indicated by red arrows. In (**g**) and (**h**), data are means of at least 3 kidneys. Error bars indicate standard errors of the mean. *p*-values were calculated using unpaired t-tests. *p < 0.05, **p < 0.008, ns; not significant. The FSK data presented are reproduced from Fig. 1.

The original Article has been corrected.

