# Supplementary material for: Targeting TRPM3 as a potential therapeutic approach for autosomal dominant polycystic kidney disease
Source: Sci Rep. 2025 Feb 8;15:4714. doi: 10.1038/s41598-025-89200-z (PMC11807189; doi:10.1038/s41598-025-89200-z)
Supplement: Supplementary file 1 — Supplementary Legends. [file 41598_2025_89200_MOESM1_ESM.docx]

**Figure S1:** Larger images of Fig 1b-g without annotation arrows.

**Figure S2:** Larger images of Fig 2b-g without annotation arrows.

**Figure S3:** Larger images of Fig 3b-g without annotation arrows.

**Figure S4:** Larger images of Fig 4b-g without annotation arrows.

**Figure S5:** Larger images of Fig 6a-f without annotation arrows.

**Figure S6:** Larger images of Fig 7a-f without annotation arrows.

**Figure S7:** IBMX induces cyst formation in a range of concentration over 10 µM and less than 100 µM (which was harmful) in E12.5 kidney rudiments. Cultured E12.5 kidneys were treated with IBMX immediately after being placed into culture and were imaged and quantified after 5 days of culture. (**a-f**) Brightfield images of E12.5 kidney rudiments treated with 0.5 µM, 1 µM, 5 µM, 10 µM, 25 µM and 50 µM IBMX. (**g, h**) Quantification of cystic areas and cyst numbers in E12.5 kidney rudiments after 5 days of IBMX treatment in increasing concentrations. Cysts were indicated by red arrows. In **g** and **h**, data are means of at least 3 kidneys and error bars indicate standard errors of the mean.

**Figure S8:** Isosakuranetin sensitized the kidneys to IBMX, in terms of cyst formation. Cultured E12.5 kidneys were treated with 20 µM isosakuranetin and varying concentrations of IBMX. Cyst formations were imaged and quantified after 5 days of culture. (**a-f**) Brightfield images of E12.5 kidney rudiments treated with 20 µM isosakuranetin and 20 µM isosakuranetin with 0.5 µM, 1 µM, 5 µM, 10 µM, 25 µM and 50 µM IBMX. (**g, h**) Quantification of cystic areas and cyst numbers in E12.5 kidney rudiments after 5 days of 20 µM isosakuranetin with varying concentrations of IBMX (maroon line) and IBMX alone (purple line). Cysts were indicated by red arrows. In **g** and **h**, data are means of at least 3 kidneys for IBMX plots and for isosakuranetin + IBMX plots. Error bars indicate standard errors of the mean. p-values were calculated using unpaired t-tests. *p< 0.04, ns; not significant.

**Figure S9:** Nifedipine attenuated IBMX-driven cyst volume. Cultured E12.5 kidneys were treated with 3µM nifedipine and varying concentrations of IBMX. Cyst formations were imaged and quantified after 5 days of culture. (**a-f**) Brightfield images of E12.5 kidney rudiments treated with 3µM nifedipine and 3µM nifedipine with 0.5 µM, 1 µM, 5 µM, 10 µM, 25 µM and 50 µM IBMX. (**g, h**) Quantification of cystic areas and cyst numbers in E12.5 kidney rudiments after 5 days of 3µM nifedipine with varying concentrations of IBMX (green line) and IBMX alone (purple line). Cysts were indicated by red arrows. In **g** and **h**, data are means of at least 3 kidneys for IBMX and for nifedipine + IBMX plots. Error bars indicate standard errors of the mean. p-values were calculated using unpaired t-tests. *p< 0.03, ns; not significant.

**Figure S10:** A comparison of the TRPM3 blocker, isosakuranetin and the TRPM3 activator, nifedipine in IBMX-driven cyst formation. Graphs illustrate (**a**) area of cysts and (**b**) number of cysts in kidneys treated with IBMX alone (purple line) IBMX with TRPM3 inhibitor (isosakuranetin + IBMX, maroon line), and IBMX with TRPM3 activator (nifedipine + IBMX, green line). Error bars indicate standard errors of the mean. The data in these figures are the same data shown individually in Figures S7, S8 and S9.
