# Supplementary material for: Targeting TRPM3 as a potential therapeutic approach for autosomal dominant polycystic kidney disease
Source: Sci Rep. 2025 Feb 8;15:4714. doi: 10.1038/s41598-025-89200-z (PMC11807189; doi:10.1038/s41598-025-89200-z)
Supplement: Supplementary file 2 — Supplementary Figures. [file 41598_2025_89200_MOESM2_ESM.pptx]

## Slide 1
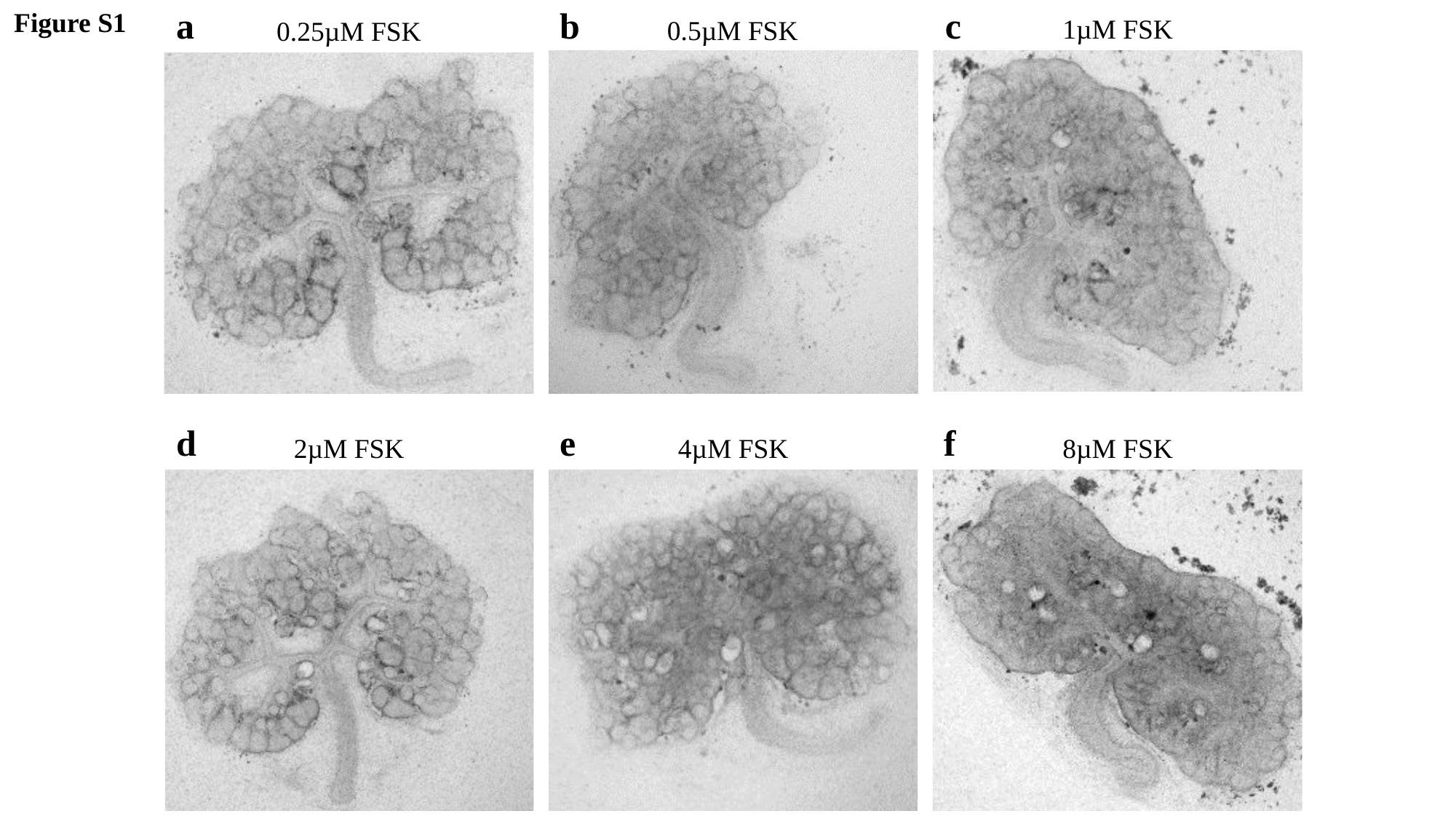

c
b
a
Figure S1
1µM FSK
0.5µM FSK
0.25µM FSK
e
f
d
4µM FSK
8µM FSK
2µM FSK

## Slide 2
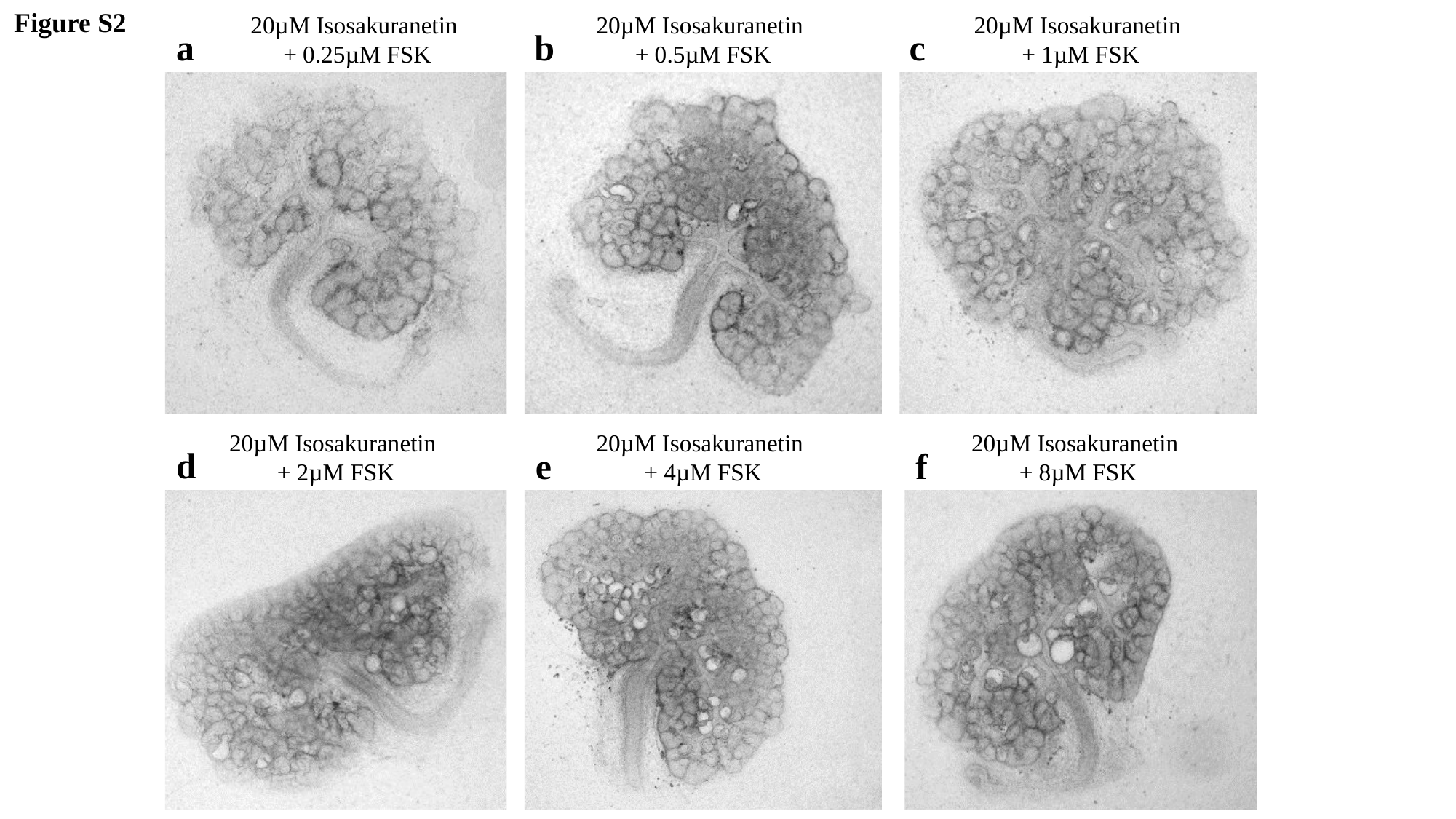

Figure S2
20µM Isosakuranetin
+ 0.25µM FSK
20µM Isosakuranetin
+ 0.5µM FSK
20µM Isosakuranetin
+ 1µM FSK
a
b
c
20µM Isosakuranetin
+ 2µM FSK
20µM Isosakuranetin
+ 4µM FSK
20µM Isosakuranetin
+ 8µM FSK
d
e
f

## Slide 3
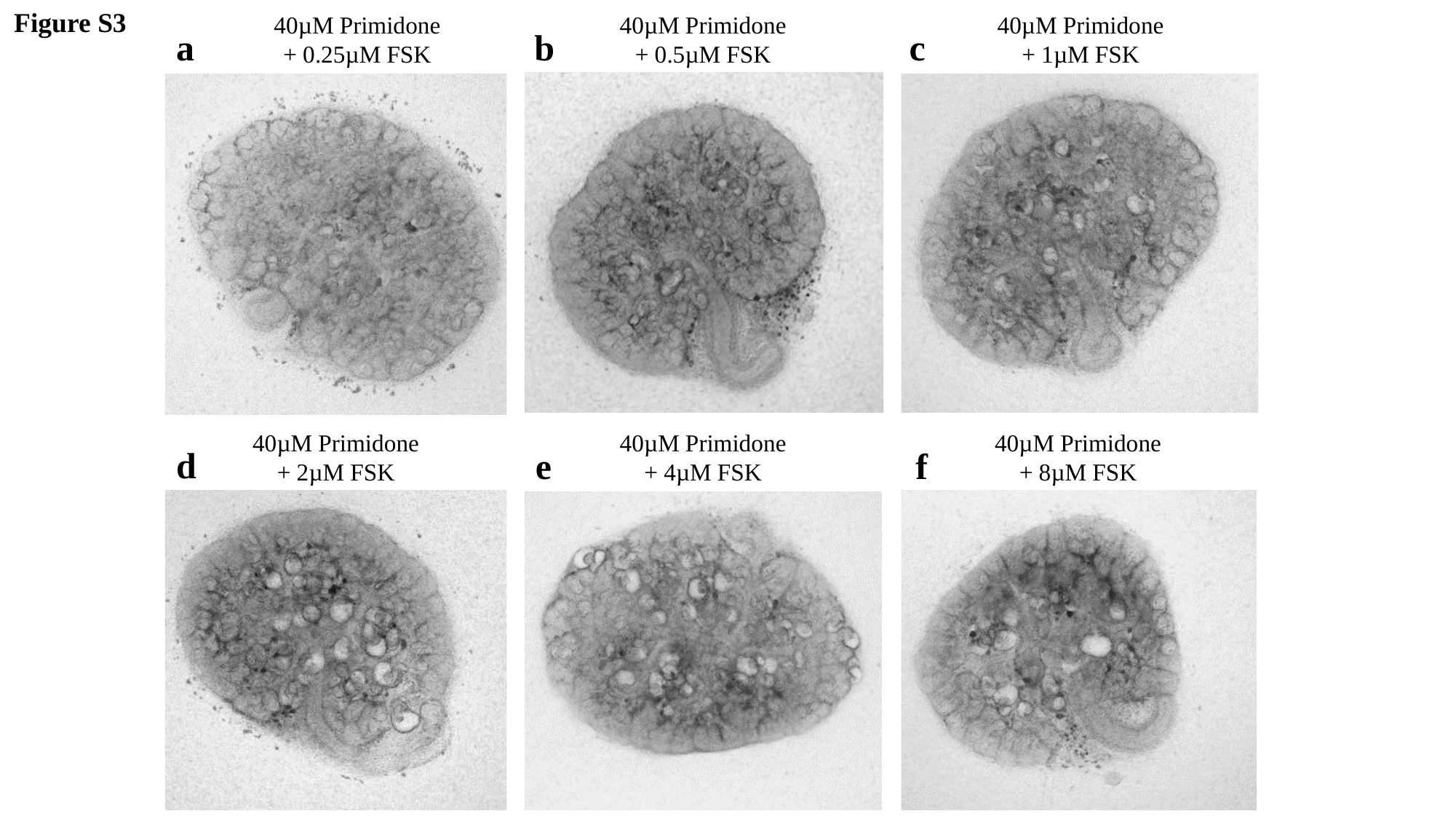

Figure S3
40µM Primidone
+ 0.25µM FSK
40µM Primidone
+ 0.5µM FSK
40µM Primidone
+ 1µM FSK
a
b
c
40µM Primidone
+ 2µM FSK
40µM Primidone
+ 4µM FSK
40µM Primidone
+ 8µM FSK
d
e
f

## Slide 4
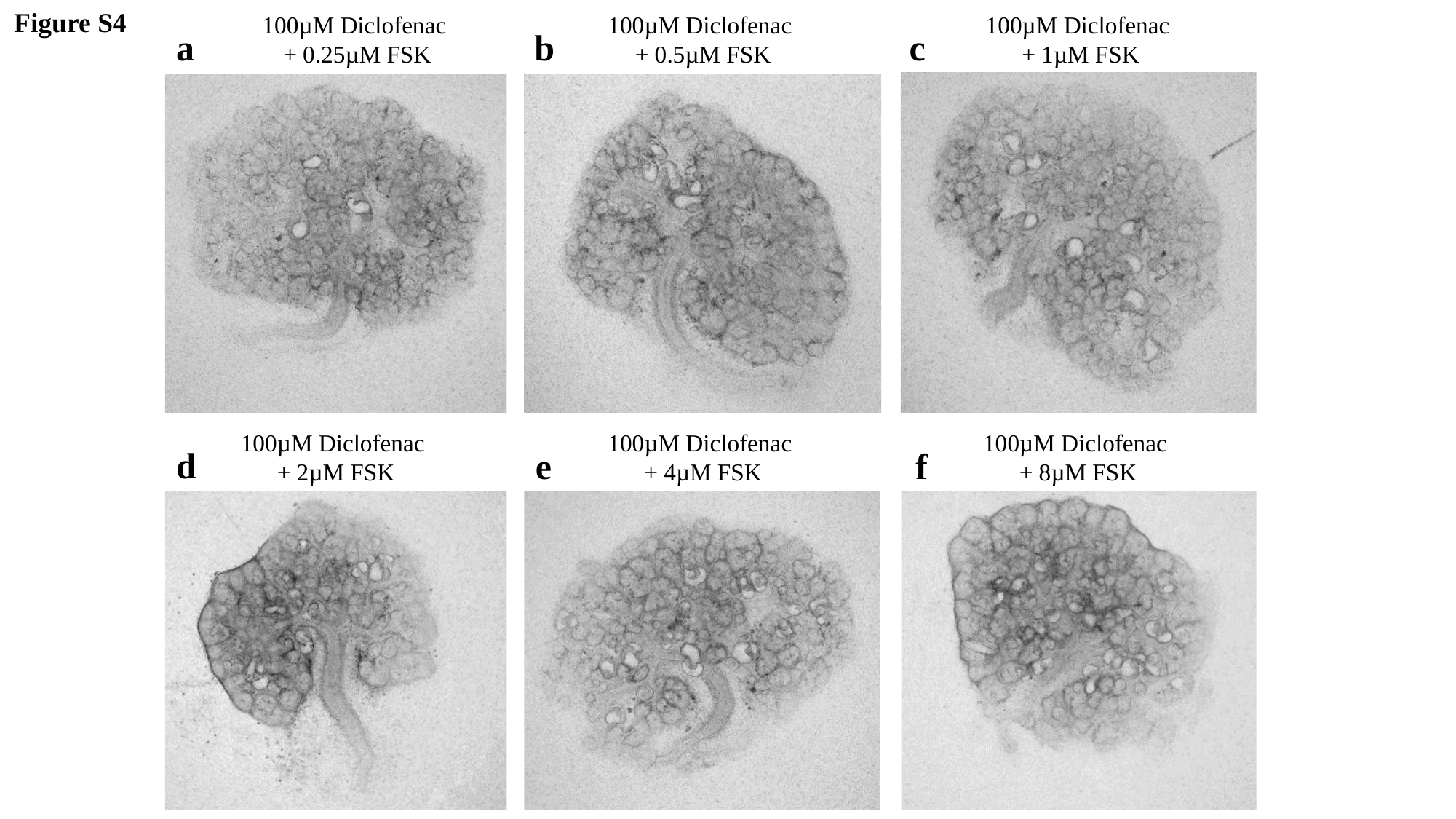

Figure S4
100µM Diclofenac
+ 0.25µM FSK
100µM Diclofenac
+ 0.5µM FSK
100µM Diclofenac
+ 1µM FSK
a
b
c
100µM Diclofenac
+ 2µM FSK
100µM Diclofenac
+ 4µM FSK
100µM Diclofenac
+ 8µM FSK
d
e
f

## Slide 5
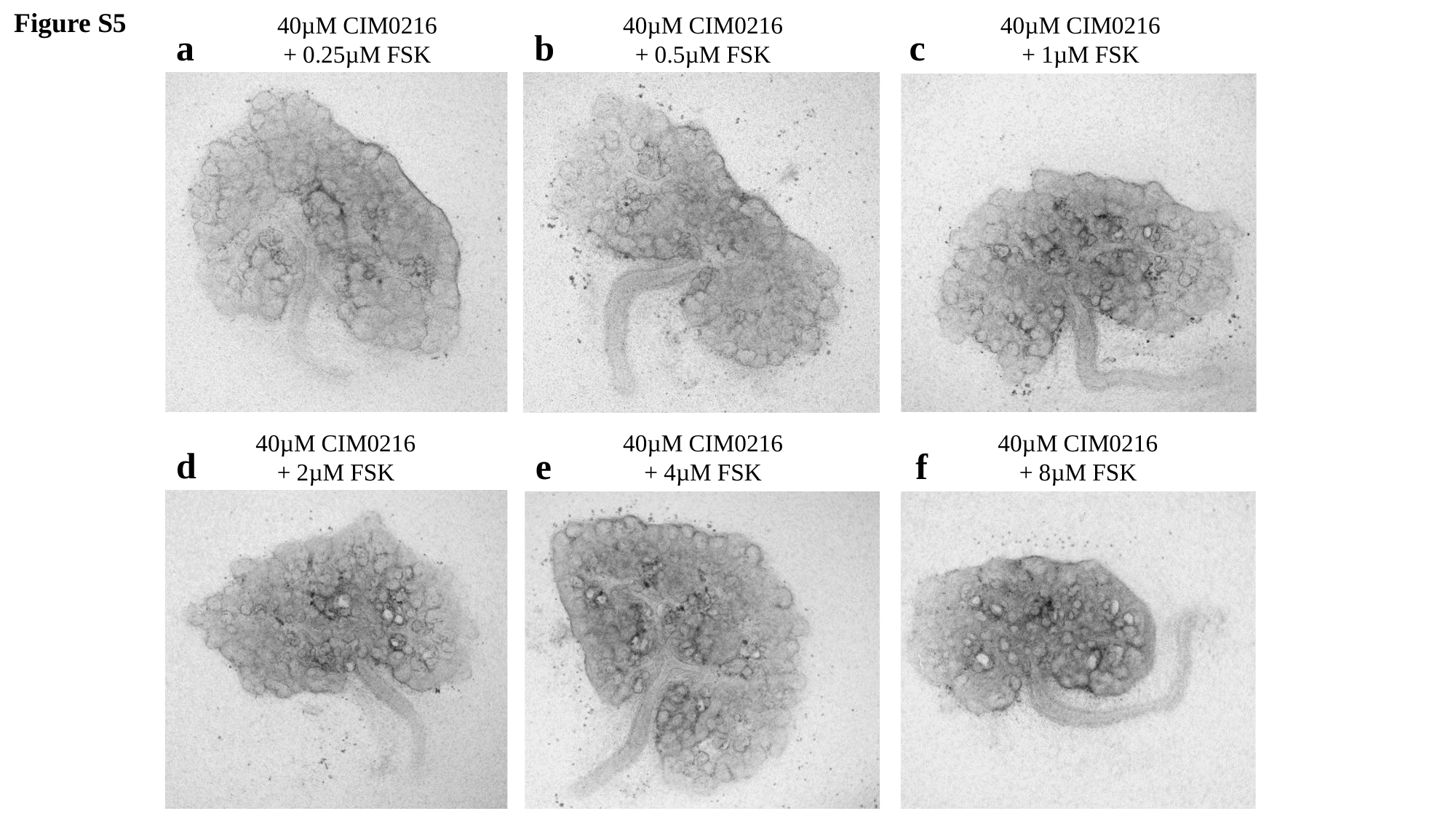

Figure S5
40µM CIM0216
+ 0.25µM FSK
40µM CIM0216
+ 0.5µM FSK
40µM CIM0216
+ 1µM FSK
a
b
c
40µM CIM0216
+ 2µM FSK
40µM CIM0216
+ 4µM FSK
40µM CIM0216
+ 8µM FSK
d
e
f

## Slide 6
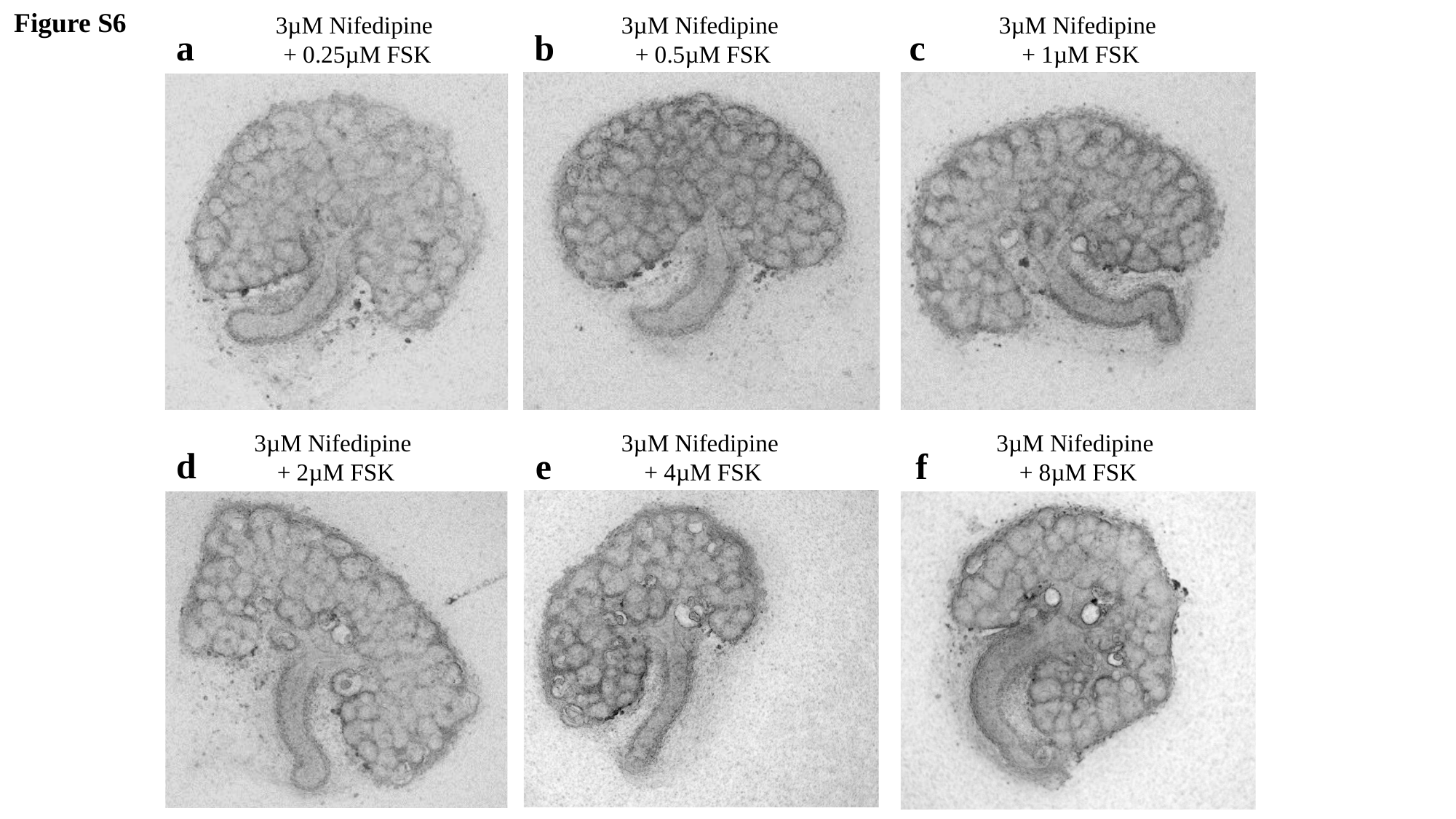

Figure S6
3µM Nifedipine
+ 0.25µM FSK
3µM Nifedipine
+ 0.5µM FSK
3µM Nifedipine
+ 1µM FSK
a
b
c
3µM Nifedipine
+ 2µM FSK
3µM Nifedipine
+ 4µM FSK
3µM Nifedipine
+ 8µM FSK
d
e
f

## Slide 7
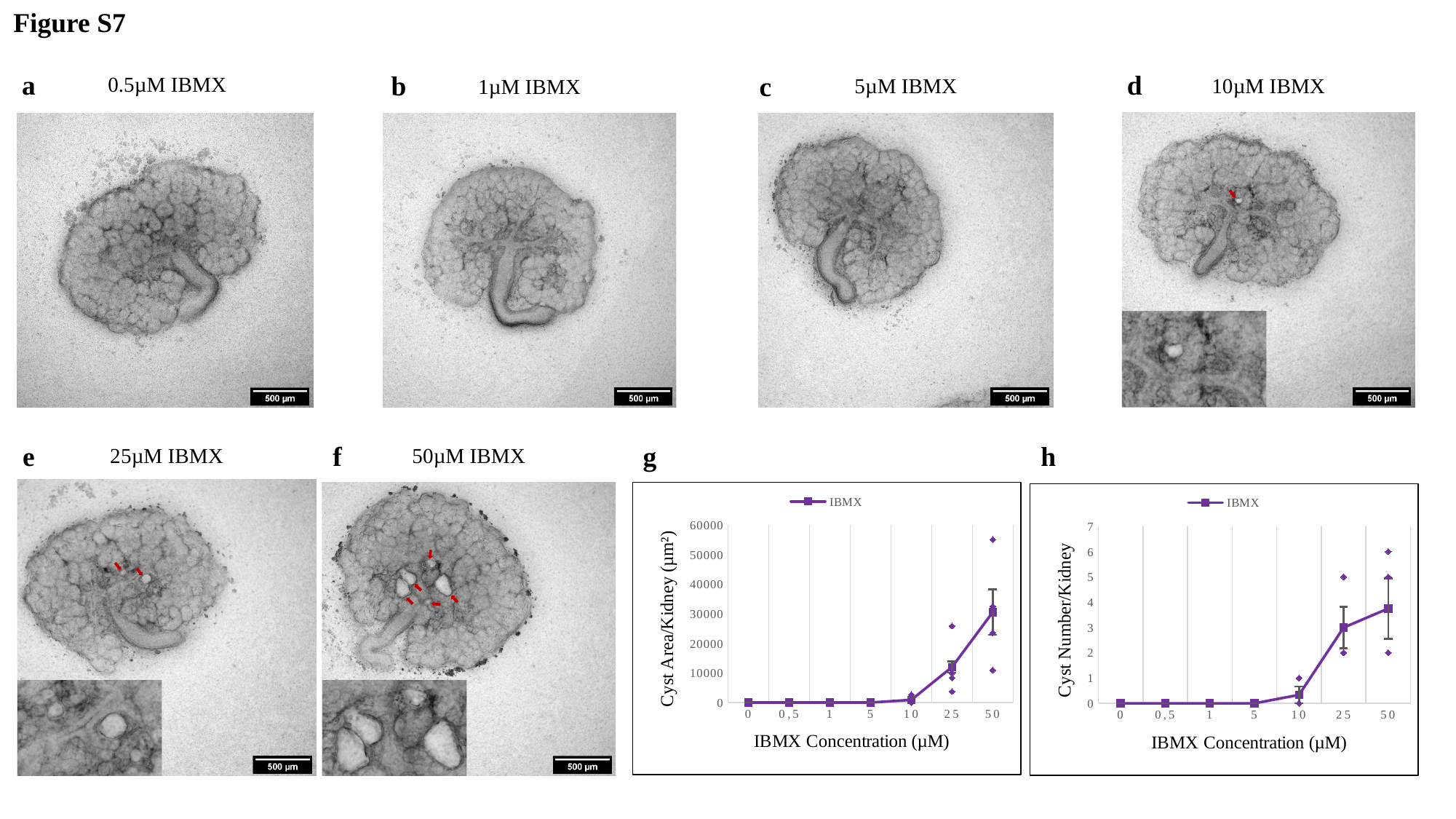

Figure S7
a
d
b
c
0.5µM IBMX
10µM IBMX
5µM IBMX
1µM IBMX
g
h
e
f
50µM IBMX
25µM IBMX
### Chart
| Category | | | | | |
|---|---|---|---|---|---|
| 0 | 0.0 | None | None | None | None |
| 0,5 | 0.0 | None | None | None | None |
| 1 | 0.0 | None | None | None | None |
| 5 | 0.0 | None | None | None | None |
| 10 | 895.172 | 2685.516 | 0.0 | None | 0.0 |
| 25 | 11980.515749999999 | 9920.828 | 3731.204 | 25899.249999999996 | 8370.780999999999 |
| 50 | 30534.867 | 32514.016 | 55128.328 | 23593.983999999997 | 10903.14 |
### Chart
| Category | | | | | |
|---|---|---|---|---|---|
| 0 | 0.0 | None | None | None | None |
| 0,5 | 0.0 | None | None | None | None |
| 1 | 0.0 | None | None | None | None |
| 5 | 0.0 | None | None | None | None |
| 10 | 0.3333333333333333 | 1.0 | 0.0 | 0.0 | None |
| 25 | 3.0 | 3.0 | 2.0 | 2.0 | 5.0 |
| 50 | 3.75 | 2.0 | 5.0 | 2.0 | 6.0 |

## Slide 8
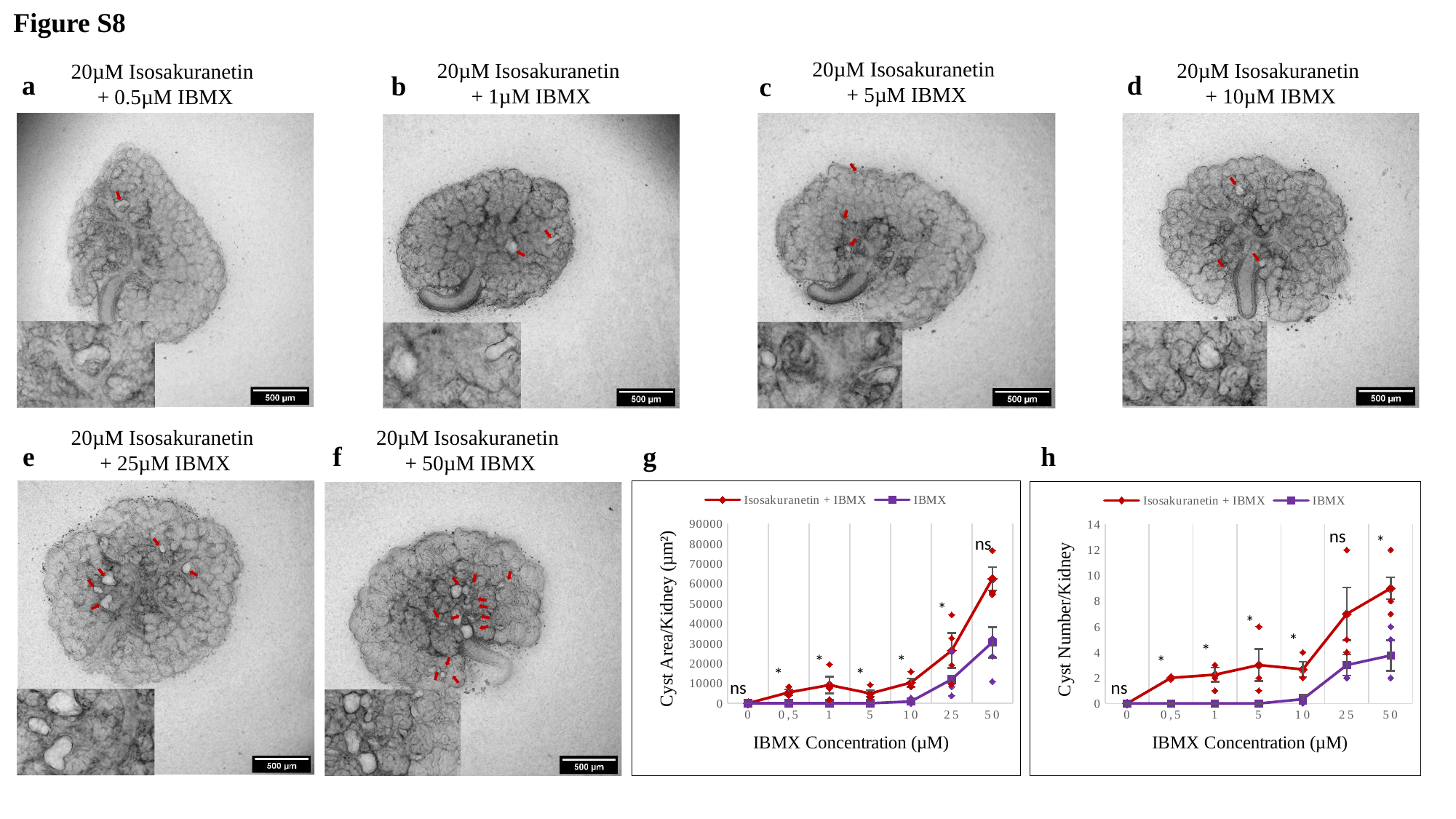

Figure S8
20µM Isosakuranetin
+ 5µM IBMX
20µM Isosakuranetin
+ 1µM IBMX
20µM Isosakuranetin
+ 10µM IBMX
20µM Isosakuranetin
+ 0.5µM IBMX
a
d
b
c
20µM Isosakuranetin
+ 25µM IBMX
20µM Isosakuranetin
+ 50µM IBMX
g
h
e
f
### Chart
| Category | | | | | | | | | | |
|---|---|---|---|---|---|---|---|---|---|---|
| 0 | 0.0 | 0.0 | None | None | None | None | None | None | None | None |
| 0,5 | 5430.0053333333335 | 0.0 | 3752.328 | 4214.438 | 8323.25 | None | None | None | None | None |
| 1 | 9129.30125 | 0.0 | 19458.766 | 1837.875 | 7840.016 | 7380.548 | None | None | None | None |
| 5 | 4910.902999999999 | 0.0 | 4230.281 | 9218.423999999999 | 3467.141 | 2727.766 | None | None | None | None |
| 10 | 10334.74625 | 895.172 | 8711.422 | 15849.030999999999 | 8278.36 | 8500.171999999999 | 2685.516 | 0.0 | 0.0 | None |
| 25 | 26389.74625 | 11980.515749999999 | 44270.078 | 32624.922000000002 | 19120.766000000003 | 9543.219 | 9920.828 | 3731.204 | 8370.780999999999 | 25899.249999999996 |
| 50 | 62414.472 | 30534.867 | 54470.810999999994 | 55857.14 | 76559.64 | 62770.297 | 32514.016 | 55128.328 | 10903.14 | 23593.983999999997 |
### Chart
| Category | | | | | | | | | | |
|---|---|---|---|---|---|---|---|---|---|---|
| 0 | 0.0 | 0.0 | None | None | None | None | None | None | None | None |
| 0,5 | 2.0 | 0.0 | 2.0 | 2.0 | 2.0 | None | None | None | None | None |
| 1 | 2.25 | 0.0 | 3.0 | 1.0 | 2.0 | 3.0 | None | None | None | None |
| 5 | 3.0 | 0.0 | 3.0 | 6.0 | 2.0 | 1.0 | None | None | None | None |
| 10 | 2.6666666666666665 | 0.3333333333333333 | 2.0 | 4.0 | 2.0 | 2.0 | 0.0 | 0.0 | 0.0 | None |
| 25 | 7.0 | 3.0 | 7.0 | 12.0 | 4.0 | 5.0 | 2.0 | 2.0 | 2.0 | 5.0 |
| 50 | 9.0 | 3.75 | 8.0 | 9.0 | 7.0 | 12.0 | 5.0 | 5.0 | 2.0 | 6.0 |
ns
*
ns
*
*
*
*
*
*
*
*
*
ns
ns

## Slide 9
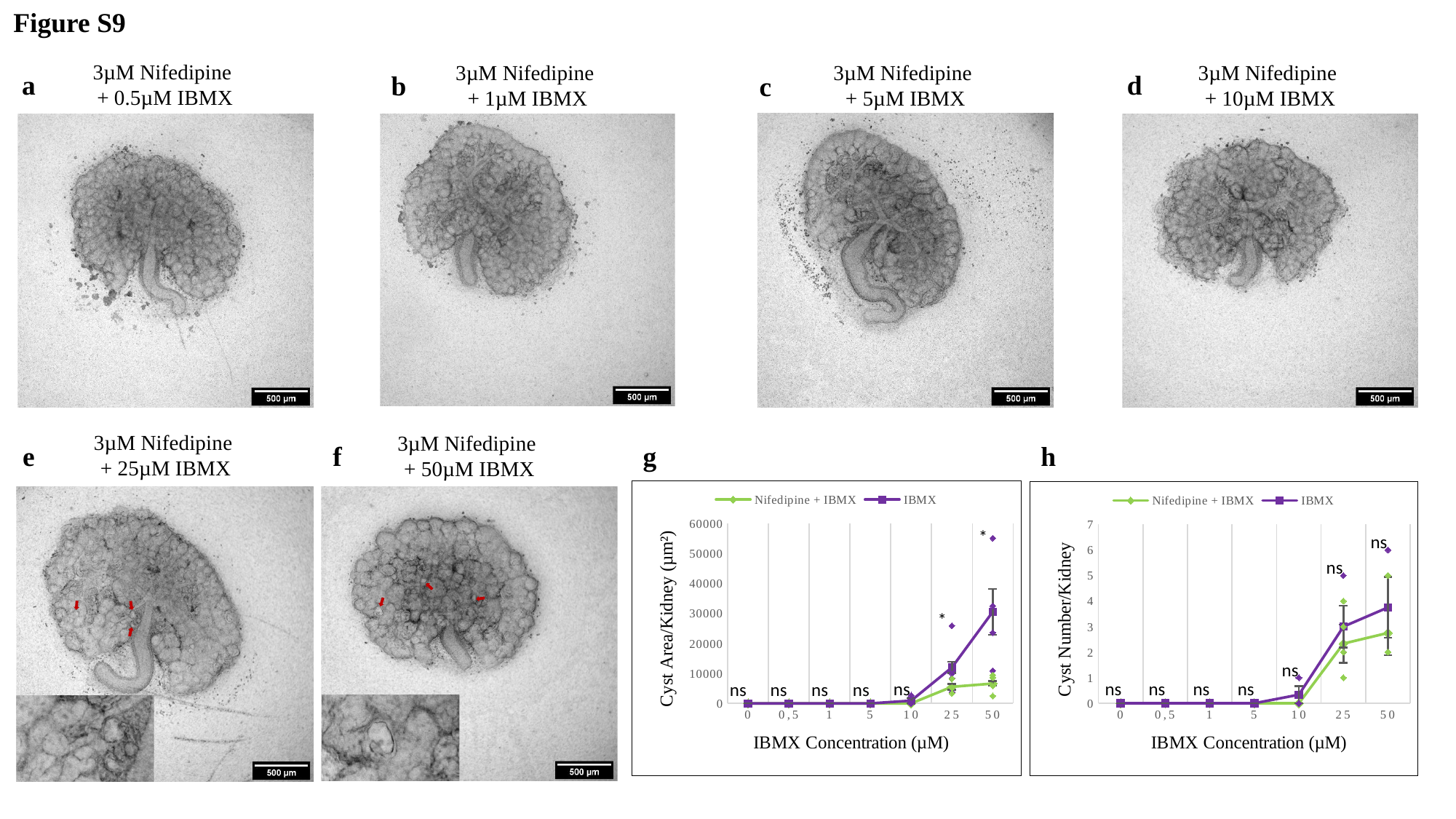

Figure S9
3µM Nifedipine
+ 0.5µM IBMX
3µM Nifedipine
+ 1µM IBMX
3µM Nifedipine
+ 5µM IBMX
3µM Nifedipine
+ 10µM IBMX
a
d
b
c
3µM Nifedipine
+ 25µM IBMX
3µM Nifedipine
+ 50µM IBMX
g
h
e
f
### Chart
| Category | | | | | | | | | | |
|---|---|---|---|---|---|---|---|---|---|---|
| 0 | 0.0 | 0.0 | None | None | None | None | None | None | None | None |
| 0,5 | 0.0 | 0.0 | None | None | None | None | None | None | None | None |
| 1 | 0.0 | 0.0 | None | None | None | None | None | None | None | None |
| 5 | 0.0 | 0.0 | None | None | None | None | None | None | None | None |
| 10 | 0.0 | 895.172 | None | None | None | None | 2685.516 | 0.0 | 0.0 | None |
| 25 | 5524.84775 | 11980.515749999999 | 3356.235 | 8244.031 | 5204.672 | 5294.453 | 9920.828 | 3731.204 | 8370.780999999999 | 25899.249999999996 |
| 50 | 6606.18375 | 30534.867 | 5885.9529999999995 | 8619.0 | 9429.671999999999 | 2490.11 | 32514.016 | 55128.328 | 10903.14 | 23593.983999999997 |
### Chart
| Category | | | | | | | | | | |
|---|---|---|---|---|---|---|---|---|---|---|
| 0 | 0.0 | 0.0 | None | None | None | None | None | None | None | None |
| 0,5 | 0.0 | 0.0 | None | None | None | None | None | None | None | None |
| 1 | 0.0 | 0.0 | None | None | None | None | None | None | None | None |
| 5 | 0.0 | 0.0 | None | None | None | None | None | None | None | None |
| 10 | 0.0 | 0.3333333333333333 | None | None | None | None | 1.0 | 0.0 | 0.0 | None |
| 25 | 2.3333333333333335 | 3.0 | 2.0 | 4.0 | 1.0 | 3.0 | 3.0 | 2.0 | 2.0 | 5.0 |
| 50 | 2.75 | 3.75 | 2.0 | 2.0 | 5.0 | 2.0 | 2.0 | 5.0 | 2.0 | 6.0 |
*
ns
ns
*
ns
ns
ns
ns
ns
ns
ns
ns
ns
ns

## Slide 10
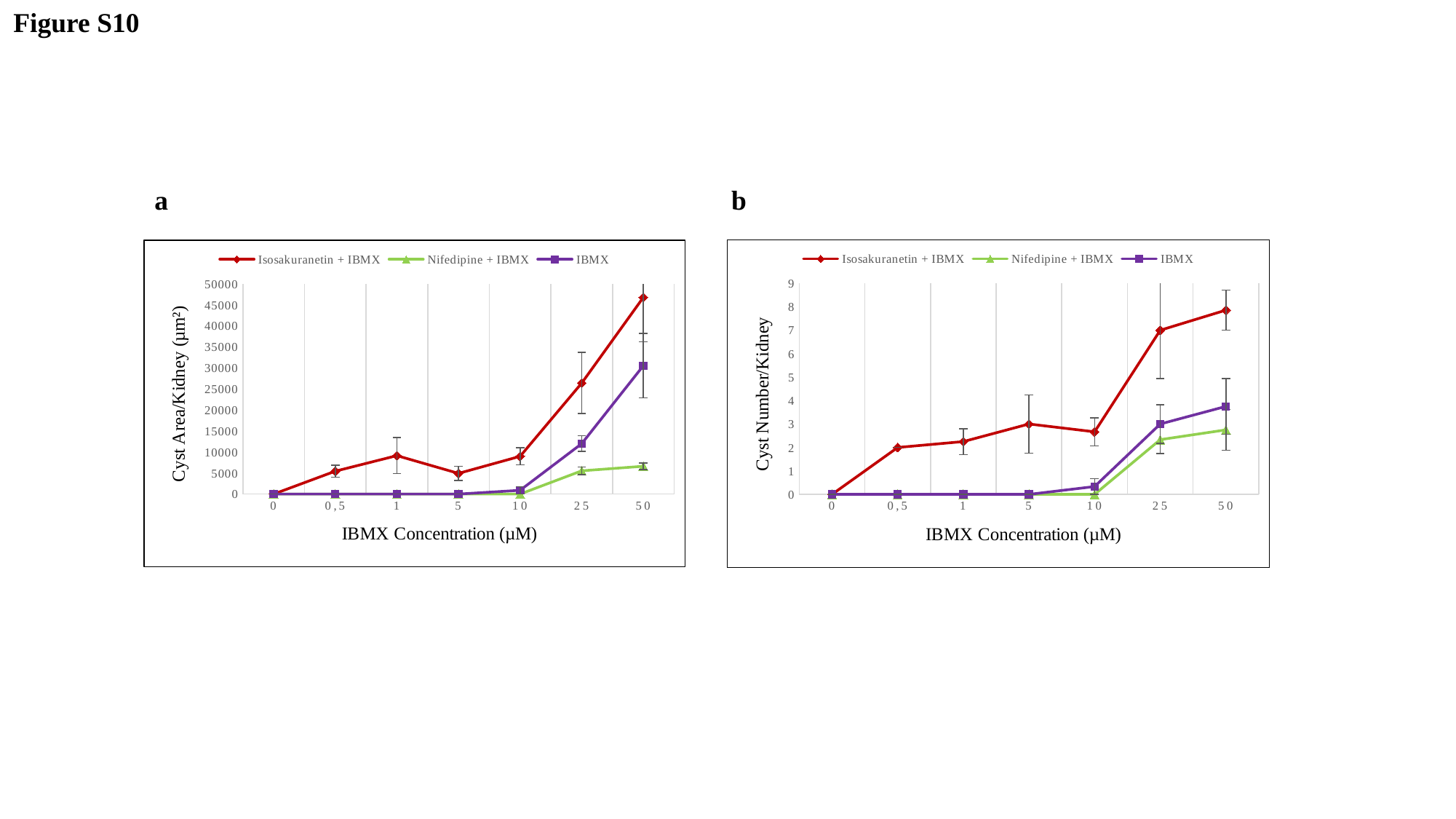

Figure S10
a
b
### Chart
| Category | Isosakuranetin + IBMX | Nifedipine + IBMX | IBMX |
|---|---|---|---|
| 0 | 0.0 | 0.0 | 0.0 |
| 0,5 | 5430.0053333333335 | 0.0 | 0.0 |
| 1 | 9129.30125 | 0.0 | 0.0 |
| 5 | 4910.902999999999 | 0.0 | 0.0 |
| 10 | 8972.844 | 0.0 | 895.172 |
| 25 | 26389.74625 | 5524.847750000001 | 11980.515749999999 |
| 50 | 46762.82771428572 | 6606.18375 | 30534.867 |
### Chart
| Category | Isosakuranetin + IBMX | Nifedipine + IBMX | IBMX |
|---|---|---|---|
| 0 | 0.0 | 0.0 | 0.0 |
| 0,5 | 2.0 | 0.0 | 0.0 |
| 1 | 2.25 | 0.0 | 0.0 |
| 5 | 3.0 | 0.0 | 0.0 |
| 10 | 2.6666666666666665 | 0.0 | 0.3333333333333333 |
| 25 | 7.0 | 2.3333333333333335 | 3.0 |
| 50 | 7.857142857142857 | 2.75 | 3.75 |
